# How Does Eye Movement Desensitization and Reprocessing Therapy Work? A Systematic Review on Suggested Mechanisms of Action

**DOI:** 10.3389/fpsyg.2018.01395

**Published:** 2018-08-13

**Authors:** Ramon Landin-Romero, Ana Moreno-Alcazar, Marco Pagani, Benedikt L. Amann

**Affiliations:** ^1^Brain and Mind Centre and School of Psychology, The University of Sydney, Sydney, NSW, Australia; ^2^ARC Centre of Excellence in Cognition and its Disorders, Sydney, NSW, Australia; ^3^Institut de Neuropsiquiatria i Addiccions, Centre Fòrum Research Unit, Parc de Salut Mar, Barcelona, Spain; ^4^IMIM (Hospital del Mar Medical Research Institute), Barcelona, Spain; ^5^Centro de Investigación Biomedica en Red de Salud Mental (CIBERSAM), Madrid, Spain; ^6^Institute of Cognitive Sciences and Technologies, CNR, Rome, Italy; ^7^Department of Psychiatry, Autonomous University of Barcelona, Barcelona, Spain

**Keywords:** eye movement desensitization and reprocessing, mechanism of action, eye movements, bilateral stimulation, systematic review

## Abstract

**Background:** Eye movement desensitization and reprocessing [EMDR] is an innovative, evidence-based and effective psychotherapy for post-traumatic stress disorder [PTSD]. As with other psychotherapies, the effectiveness of EMDR contrasts with a limited knowledge of its underlying mechanism of action. In its relatively short life as a therapeutic option, EMDR has not been without controversy, in particular regarding the role of the bilateral stimulation as an active component of the therapy. The high prevalence of EMDR in clinical practice and the dramatic increase in EMDR research in recent years, with more than 26 randomized controlled trials published to date, highlight the need for a better understanding of its mechanism of action.

**Methods:** We conducted a thorough systematic search of studies published until January 2018, using PubMed, ScienceDirect, Web of Knowledge and Scopus databases that examined the mechanism of action of EMDR or provided conclusions within the framework of current theoretical models of EMDR functioning.

**Results:** Eighty-seven studies were selected for review and classified into three overarching models; (i) psychological models (ii) psychophysiological models and (iii) neurobiological models. The evidence available from each study was analyzed and discussed. Results demonstrated a reasonable empirical support for the working memory hypothesis and for the physiological changes associated with successful EMDR therapy. Recently, more sophisticated structural and functional neuroimaging studies using high resolution structural and temporal techniques are starting to provide preliminary evidence into the neuronal correlates before, during and after EMDR therapy.

**Discussion:** Despite the increasing number of studies that published in recent years, the research into the mechanisms underlying EMDR therapy is still in its infancy. Studies in well-defined clinical and non-clinical populations, larger sample sizes and tighter methodological control are further needed in order to establish firm conclusions.

## Introduction

While the methodology that guides the Eye Movement Desensitization and Reprocessing [EMDR] intervention has been clinically validated, its mechanism of action remains elusive. Since the early 90's, different speculative theories, models and hypotheses have been proposed (with ever growing sophistication) to explain the neurobiological underpinnings of EMDR. Furthermore, the growing popularity of EMDR as evidenced by the increasing number of studies available in research databases, suggests that a systematic review is timely. Finally, the implementation of EMDR in clinical practice before unraveling its mechanism of action has motivated stark criticism by some authors (Herbert et al., 2000).

The current manuscript have two main aims. The first aim is to provide an overview of the development of EMDR over the last 25 years, including the procedural aspects of EMDR and current controversies about its efficacy. The second aim is to conduct a systematic review of the theoretical hypotheses and available empirical evidence regarding the mechanism of action of EMDR.

### The development of eye movement desensitization and the first study

The year 2014 marked the 25th anniversary of the introduction of EMDR, a relatively novel psychotherapy now well-established and recognized internationally as an empirically supported treatment for trauma. The American psychologist Francine Shapiro first developed EMDR upon her chance observation while walking through a park that certain saccadic eye movements [EMs] reduced the intensity of disturbing thoughts. She then noticed that bringing the EMs under voluntary control while thinking about a distressing memory reduced the anxiety associated to it. Shapiro then conducted a randomized controlled trial in which she administered one session of eye movement desensitization [EMD] to 22 patients suffering from traumatic memories (Shapiro, 1989a,b). The results of this study indicated that EMD successfully desensitized traumatic memories and decreased anxiety levels in traumatized subjects when compared to a control group that received a procedure similar to flooding. This effect was followed by a significant improvement in the negative cognitions associated with the traumatic memories, characterized by an increase in the appraised validity of a positive self-belief. These results were further maintained after 1 and 3 months of follow-up.

### From EMD to EMDR: the standard EMDR therapy protocol

Shapiro's initial studies supported the hypothesis that EMs facilitated the desensitization of trauma memories (Shapiro, 1989a). In subsequent years, EMD grew into EMDR in recognition of its hypothesized memory reprocessing effects, and evolved toward a structured eight-phase approach using standardized procedures to address the past, present, and future aspects of a traumatic memory (Shapiro, 2001). The traumatic memory is composed of a set of multi-sensory images, negative cognitions, negative emotions, and related unpleasant physical sensations. The EMDR therapy standard protocol includes the following preparation steps: history and treatment plan [Phase I], preparation phase with an introduction to the EMDR protocol and development of coping strategies [Phase II], and an assessment phase with visualization of an image of the traumatic incident, identification of beliefs and emotions associated with the disturbing event, rating of disturbance recalling the traumatic incident, and rating the validity of preferred cognitions of the client (Phase III). The desensitization and reprocessing takes place within Phase IV and represents the core component of the intervention: the client focuses on a dual attention stimulus - generally eye movements- while holding in mind the image, thoughts and/or sensations associated with the disturbing memory. Bilateral tactile taps or auditory tones are used instead of eye movements for clients who have difficulty in visual tracking. Following each brief set of bilateral stimulation (BLS), the client is asked to identify the associative information that was elicited. Following standardized procedures, this new material usually becomes the focus of the next set. BLS is also used during Phase V, which aims to incorporate and strengthen a positive cognition to replace the negative cognition associated with the trauma, as well as in Phase VI which entails the body scan to reprocess any remaining bodily sensations. In Phase VII the client is guided through relaxation techniques designed to re-establish emotional stability if distress has been experienced, and for use between sessions. Finally, the phase of re-evaluation [Phase VIII] involves identifying outcomes from the prior session. At this point, the therapist will decide whether it is best to continue working on previous targets or continue with newer ones. The length of an individual treatment session is typically 50–90 min, and single memories are typically processed within one-to-three sessions. Based on feedback from clinicians and patients alike, the completion of the EMDR standardized protocol is a cognitively demanding task and requires attention, self-consciousness, autobiographical semantic memory, and metacognition to successfully identify the potential dysfunctional processes underlying the traumatic memory.

### Evidence for the efficacy of EMDR in PTSD and in other comorbid mental disorders

In spite of initial controversies, the efficacy of EMDR treatment for PTSD is now well documented (e.g., Shepherd et al., 2000; Davidson and Parker, 2001; Bradley et al., 2005; Novo Navarro et al., 2016). Since the original observation of Shapiro, over 300 studies have examined the clinical application of EMDR and several meta-analyses have shown higher or similar efficacy in PTSD compared to pharmacological or other psychological interventions (Born et al., 2006; Bisson et al., 2007, 2013; Chen et al., 2014). EMDR is now recognized by the National Institute for Health and Clinical Excellence (Born et al., 2005) and the World Health Organization (Born et al., 2013) as a treatment of choice for post-traumatic stress disorder. The accumulating evidence on how trauma and life events–adverse or not–can become causal factors in the etiology of different psychological disorders (Lytle et al., 2002; Christman et al., 2003; Lohr et al., 2003; Taylor et al., 2003; Van Loey and Van Son, 2003) is motivating clinicians and practitioners to offer EMDR as a comprehensive therapy for different conditions, regardless of whether there is evidence of diagnosis of PTSD, or comorbid traumatic memories. As such, evidence for a variety of EMDR therapy applications has recently been reported in randomized controlled trials of bipolar disorder (Novo et al., 2014; Moreno-Alcázar et al., 2015), psychosis (van den Berg et al., 2015a,b), unipolar depression (Hase et al., 2015), dental phobia (Doering et al., 2013), obsessive compulsive disorder (Nazari et al., 2011), panic disorder (Faretta, 2012), alcohol dependency (Perez-Dandieu and Tapia, 2014), and pain management (Tesarz et al., 2014).

### The adaptive information processing model

The Adaptive Information Processing (AIP) model is the theory that guides the EMDR treatment procedures and offers an explanation for the basis of pathology (Shapiro, 1994, 2001, 2007). This model postulates that humans have an innate information processing system that assimilates new experiences and stores them into existing memory networks in an adaptive state. These networks link the thoughts, images, emotions, and sensations associated with experiences. According to the AIP model, pathology arises when new information is inadequately processed and then stored in a maladaptive mode in the memory networks, along with associated distorted thoughts, sensations and emotions. Thus, external stimulation similar to the adverse experience can trigger sensations and images from the traumatic event so that the person re-experiences feelings or bodily sensations. If these memories remain unprocessed, they become the basis of the symptoms of PTSD. Conversely, AIP theory hypothesizes that when the memories are adequately processed, symptoms can be eliminated and integrated. Shapiro proposed that EMDR can assist in processing the traumatic memories, and that different forms of bilateral stimulation such as the EMs, would facilitate this processing (Shapiro, 2001; Shapiro and Maxfield, 2002).

### Controversies surrounding EMDR therapy

Since its inception, EMDR has generated a considerable debate, particularly regarding the role of the EMs as an active ingredient of treatment. Similarly, there is ongoing controversy on whether the underlying mechanisms in EMDR differ substantially from those operating in trauma-focused cognitive-behavioral therapy [tfCBT] and standard exposure.

The use of a dual attention tasks is perhaps one of the most distinctive elements of EMDR. As described above, this involves the client focusing on the worst image of a traumatic memory while concurrently engaging in an external task, typically following the therapist's fingers using rhythmic, bilateral, saccadic EMs. The EMs were originally described as the “crucial component” of EMDR (Shapiro, 1989a,b). Some studies are suggestive of a unique contribution of the EMs to successful treatment (Andrade et al., 1997; Kavanagh et al., 2001; van den Hout et al., 2001; Lee and Drummond, 2008), while others have not find clear differences in the outcome comparing EMDR with and without EMs (Cahill et al., 1999; Davidson and Parker, 2001). Head-to-head comparison between the results of these early studies is not possible as they differ considerably in terms of design, samples and outcome measures. Therefore, some authors argue that the claims of no significant effect of the EMs on treatment outcome are unwarranted (Jeffries and Davis, 2013). In recent years, studies have found accumulating evidence on the contribution of BLS (and in particular the EMs) to treatment gains, including a meta-analysis of 26 randomized controlled trials that found a significant contribution of the EMs in processing emotional memories (Lee and Cuijpers, 2013). Research has also found that other forms of BLS, such as bilateral tactile taps or auditory tones, are also effective methods of reducing vividness in trauma (van den Hout et al., 2011b; de Jongh et al., 2013). This evidence led Shapiro to conclude that dual attention may be the mechanism responsible for the treatment gains rather than any effect unique to the EMs (Shapiro and Laliotis, 2015).

A second contentious issue in EMDR revolved around the potential overlap with other psychotherapies, in particular with tfCBT. While tfCBT consists of exposure techniques combined with cognitive interventions, EMDR is an eclectic form of psychotherapy that incorporates structured procedures and protocols. Although many of the EMDR procedures appear to overlap with tfCBT, the UK National Institute of Health and Clinical Excellence [NICE] has stated that these two approaches are different since specific training programs are required [NICE, 2005, p. 55]. Like tfCBT, EMDR aims to reduce subjective distress and strengthen adaptive cognitions related to the traumatic event. Unlike tfCBT, EMDR does not involve (i) detailed descriptions of the event, (ii) direct challenging of beliefs, (iii) extended exposure, or (iv) homework. Rogers and Silvers have described in detail the differences between how exposure (a key component of tfCBT) and EMDR protocols are employed (Rogers and Silver, 2002). Evidence has grown in recent years that EMDR therapy produces diverse and compelling treatment effects, including a reconsolidation of memory structures through mechanisms that differ from those of traditional exposure therapy (Lee et al., 2006; Ecker et al., 2012). Ultimately, the debate on the overlap between EMDR and tfCBT is flawed, at least in terms of their underlying mechanisms of action, given the limited knowledge of the impact of different psychotherapies on neurobiological changes associated with PTSD and other anxiety disorders.

### Objectives and importance of the current review

Previous systematic reviews and meta-analyses of EMDR have been limited to specific elements and hypotheses or were non-systematic in nature (Gunter and Bodner, 2009; McGuire et al., 2014). Some examples of this are reviews focusing on the effect of the EMs on the therapy (Jeffries and Davis, 2013; Lee and Cuijpers, 2013), and on the physiological (Elofsson et al., 2008) and the neurobiological correlate of EMDR (Bergmann, 2008; Pagani et al., 2013). In the current work, we have conducted a comprehensive review of the literature that examined different hypothesis for the mechanism of action of EMDR using the PRISMA guidelines for transparent reporting of reviews and meta-analyses. PRISMA is an evidence-based minimum of 27 items grounded on evidence that establishes the minimum criteria for reporting systematic reviews. Although it focuses on reporting reviews of randomized controlled trials, it can also be used as a basis for reporting systematic reviews of other types of research (Moher et al., 2009).

## Methods

Studes examinig the mechanism of action of EMDR were identified using PubMed, ScienceDirect, Web of Knowledge and Scopus databases. The systematic literature search included studes published from 01/01/1989 until 31/12/2017 based on the PRISMA guidelines (Supplementary Data Sheet). The search terms were selected from the thesaurus of the National Library of Medicine (Medical Subject Heading Terms, MeSH) and the American Psychological Association (Psychological Index Terms) and included the terms “eye movement desensitization and reprocessing,” “EMDR,” “mechanism,” “action,” “effects,” and “correlates.” The final search equation was defined using the Boolean conectors “AND” and “OR” following the formulation: (“eye movement desensitization and reprocessing” OR “EMDR”) AND (“mechanism” OR “action” OR “effects” OR “correlates”). The automatic search was later completed with a manual search using reference lists of included papers and web-based searches in an EMDR-centered library (https://emdria.omeka.net/). Titles, abstract, methods and results of the articles identified were screened for pertinent information. Reference lists of eligible articles and relevant review articles were also screened for potential publications for inclusion. The search did not include any subheadings ot tags (i.e., search fields “All fields”). Due to the significant heterogeneity of the studies, a formal quantitative synthesis (i.e., meta-analysis) was not possible. Instead, a systematic review was conducted, using the PRISMA guidelines as referenced above.

### Inclusion criteria and exclusion criteria

The final selection of research articles was conducted using the following criteria: (i) original articles published in peer-reviewed journals, (ii) in adult populations that (iii) examined the mechanism of action of EMDR and/or (iv) any form of BLS (EM, tactile, sound) within the EMDR protocol or (v) provided conclusions regarding the potential mechanism of action of EMDR. Selected theoretical, speculative papers were also included if they were first to provide an mechanistic hypothesis for EMDR to guide future empirical research. The criteria for exclusion were: (i) articles that did not contain original research (i.e., reviews and meta-analyses, guidelines and/or protocols), (ii) clinical trials and/or focus on treatment gains or efficacy and (iii) empirical studies with quasi-experimental designs (single case and/or no control group). The studies were selected by RL-R and AM-A. Discrepancies were resolved by MP and BLA (Supplementary Table 1).

## Results

**Figure 1:**
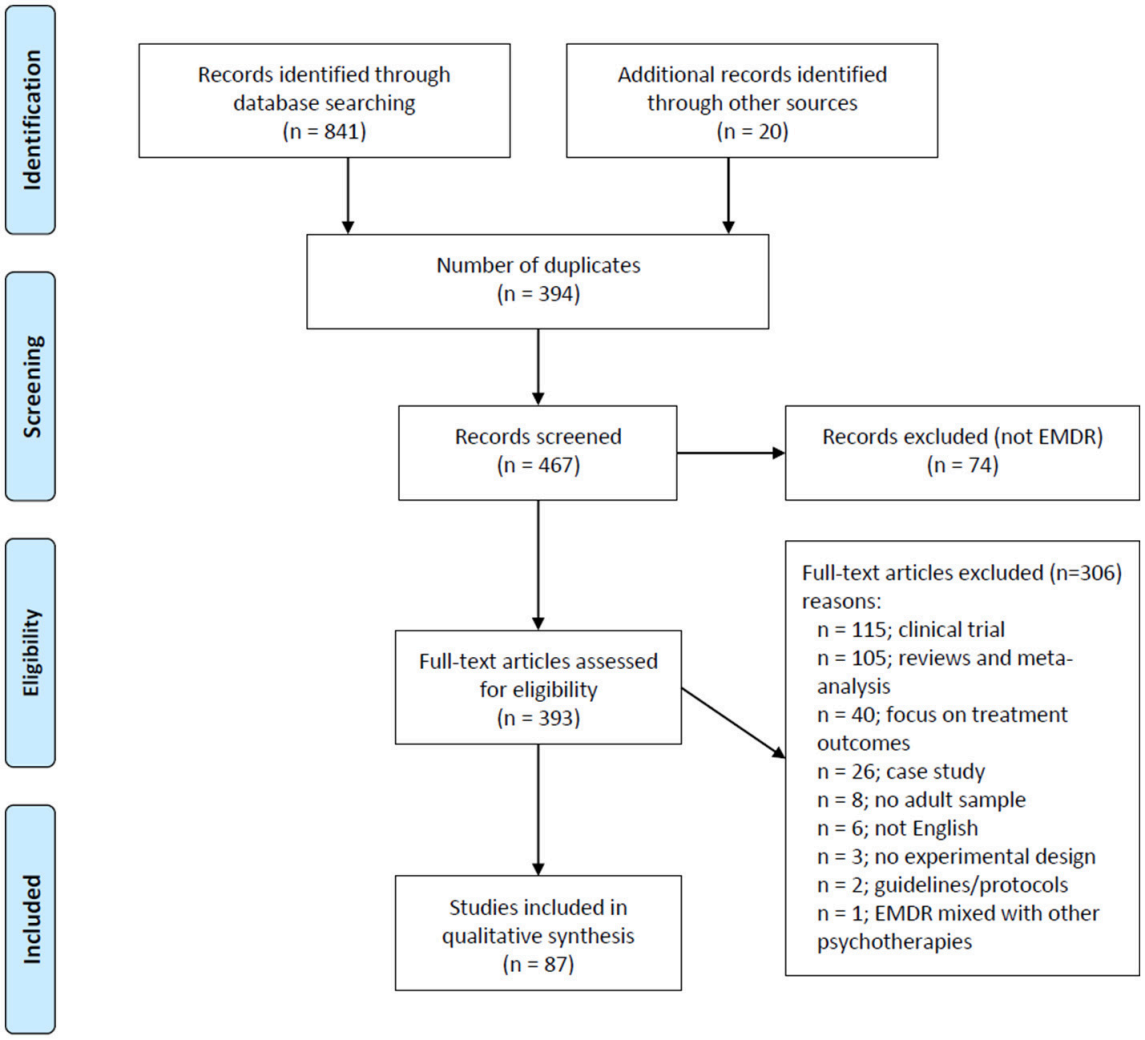
Flow chart for the selection of eligible studies.

Figure 1 shows a flow-chart for the selection of eligible studies. The search strategy initially identified 841 studies thorugh database searching and 20 additional studies through manual searches in other sources (i.e., Shapiro Libray). After removing duplicates (*n* = 394), RL-R, and AM-A screened titles and abstracts and excluded studies that were considered non-pertinent (*n* = 74). If inclusion criteria were met, the full text article was retrieved and screened in full for the analysis.

A total of 87 studies written in English met the inclusion criteria and were selected for review. The studies were classified into broad categories according to three overarching models/hypothesis for the mechanism of action underlying EMDR: (i) psychological models (ii) psychophysiological models and (iii) neurobiological models. A summary of the main characteristics of each study, including participants, methods, sample size, control conditions, study design, outcomes and conclusions can be gathered from Tables 1–3.

**Table 1:** Psychological models (*n* = 32).

**Author, year**	**Type of study**	**Sample (*n*)**	**EM/Full protocol**	**Control condition**	**Main findings**	**Conclusions**
**THE ORIENTING AND RELAXATION RESPONSE**						
Dyck, 1993	Speculative theory	NA	NA	NA	NA	Classic conditioning theory as a framework for the effects of EM in traumatic memories.
Armstrong and Vaughan, 1996	Speculative theory	NA	NA	NA	NA	The EM trigger an OR that facilitates attention to the trauma memory without avoidance.
MacCulloch and Feldman, 1996	Speculative theory	NA	NA	NA	NA	Combination of Pavlovian and Darwinian theory to explain the effectiveness of EMDR. Positive elements of the OR are paired unpleasant memories to remove their negative effect.
Wilson et al., 1996	Empirical study	HC (*n* = 18)	Full protocol	Full protocol with no EM Tapping	EMDR group showed desensitization. Autonomic changes during EMDR compatible with a relaxation response.	The EMDR therapeutic effect is provoked by pairing distress with an unlearned relaxation response.
Kuiken et al., 2010	Empirical study	HC (*n* = 101)	EM	No EM	Rapid bilateral EM activate the orienting response and, by doing so, facilitate attention to and comprehension of figurative, especially metaphoric, expressions.	Rapid EM in the EMDR protocol prompt novel shifts in memory (e.g., diminution of threat), belief (e.g., recognizing unintentional responsibility), and emotion (e.g., changing fear to anger).
**THE WORKING MEMORY ACCOUNT**						
Sharpley et al., 1996b	Empirical study	HC = 24	EMDR	Rapid Induction Relaxation	EMDR reduced the vividness more significantly vs. control conditions.	EMDR reduces the vividness of a memory-based image.
Andrade et al., 1997	Empirical study	Exp 1:HC = 46 Exp 2: HC = 18 Exp 3: HC = 30 Exp 4: HC = 24	EM	Fixed eyes Tapping No dual task	EMs reduced vividness and emotiveness of trauma vs. control conditions.	EMDR effects are mediated by the visuospatial sketchpad of working memory.
Kavanagh et al., 2001	Empirical study	HC = 18	EM	Visual noise Exposure alone	EMs reduced vividness and emotiveness of trauma vs. control conditions.	A visuospatial task (e.g. EMs) offer a temporary response aid for imaginal exposure without affecting desensitization.
van den Hout et al., 2001	Empirical study	HC = 60	EM	Finger tapping No dual task	EMs reduced vividness of positive and negative recollections.	The effect of EMs is mediated by VSSP taxation.
Gunter and Bodner, 2008	Empirical study	Exp 1: HC = 36 Exp 2: HC = 36 Exp 3: HC = 72	EM	Stationary eyes Horizontal EMs Auditory shadowing Drawing	Vertical and horizontal EMs reduce vividness and increase arousal.	The central executive of the WM is taxed when a person performs a distractor task while attempting to hold a memory in mind.
Maxfield et al., 2008	Empirical study	Exp 1: HC = 24 Exp 2: HC = 36	EM	No EM Slow EM Fast EM	Fast EMs produce significant decrease of emotional intensity.	The decrease of emotional intensity is mediated by competition for WM resources
Lilley et al., 2009	Empirical study	HC = 18	EM	Counting No concurrent task	EMs reduces vividness and emotionality.	Concurrent tasks matched to the modality of trauma images lessening emotional responses to recollections of trauma.
van den Hout et al., 2011b	Empirical study	HC = 15	EM	Bilateral “beeps”	EMs slow down reaction times to auditive cues.	The effect of beeps on taxing negative memories are inferior to those of EMs.
Kristjánsdóttir and Lee, 2011	Empirical study	HC = 36	EM	Counting	Vividness and emotionality significantly decreased after EMs and counting, with EMs producing the greatest effect irrespective memory modality.	Results are consistent with the taxation of the central executive of WM.
van den Hout et al., 2012	Empirical study	PTSD = 12	EM	Beeps Recall only	EMs are better than tones in reducing vividness. Tones are better than recall only.	Results support for WM model. Tones may outperform EMs in cases where trauma memories are vague.
Smeets et al., 2012	Empirical study	HC = 61	EM	Eyes stationary	EMs outperformed eyes stationary condition in reducing vividness first and then emotionality.	Emotionality is reduced only after vividness has dropped.
van den Hout et al., 2013	Empirical study	HC = 32	EM	Eyes stationary	In the EM group, self-rated vividness of the recalled+EM picture decreased, relative to the non-recalled picture. In the no-EM group there was no difference between the recalled versus non-recalled picture.	Reduction of memory vividness due to recall+EM is also evident from non-self-report data.
Novo Novo Navarro et al., 2013	Empirical study	HC = 50	EM	Eye rest condition	No significant differences between EM and fixed eye condition in recall.	EM did not improve auditory and visual consolidation of memory, undermining this WM taxing as a mechanism of action of EMDR
de Jongh et al., 2013	Empirical study	PTSD = 32 Other mental disorder = 32	EM	Tones Eye rest condition	Effects of EMs >tones > recall only.	EM effects of taxing WM on disturbing memories do no differ between PTSD and other metal disorders.
Leer et al., 2014	Empirical study	HC = 73	Recall with EM	Recall only	Recall with EM decrease vividness vs. recall only.	Recall with EM causes 24-h changes in memory vividness/emotionality.
van den Hout et al., 2014	Empirical study	HC = 40	Recall with EM	Recall only	Negative memories are rated as less vivid after “recall + EM” but not after “recall only”. This was not found for neutral memories.	Emotional memories are more taxing than neutral memories.
Leer et al., 2017	Empirical study	HC (*n =* 26)	EM	Recall with no EM	EM slow down reaction time in a stimulus discrimination task.	EM during recall attenuates memory performance and renders stimulus attributes less accessible
van Veen et al., 2016	Empirical study	HC (*n =* 108)	EM	Recall with no EM	EM showed a larger decrease in self-reported vividness and emotionality than control conditions.	Recall of an aversive memory loads working memory but drops in vividness and emotionality do not immediately reduce the cognitive load of recalling the memory
van Schie et al., 2016	Empirical study	HC (*n =* 66)	EM	recall + slow EM, and recall + fast EM	Speed differences of EM do not affect recall. Cognitively demanding dual task increases the intervention's effectiveness.	Adjusting EM speed is not helpful to reduce emotionality of aversive memories.
van Veen et al., 2015	Empirical study	HC (*n =* 106)	EM	recall + fast EM, recall + slow EM, or recall only	recall + fast EM led to less emotional, less vivid and more difficult to retrieve images than recall + slow EM and recall only.	Results support the WM theory: the more taxing a dual-task is, the more a memory image degrades
Engelhard et al., 2010a	Empirical study	HC = 28	EM	Exposure	EMs reduce vividness of past and future feared events.	Taxing of WM provokes degradation of visual imagery about feared future events.
Engelhard et al., 2010b	Empirical study	HC = 60	EM	Tetris game	EMs and Tetris draw on WM, vs. a no dual-task. Compared to recall only, EM and Tetris both decreased emotionality.	Both EMs and Tetris tax WM.
Engelhard et al., 2011	Empirical study	HC = 37	EM	Stationary eyes recall	Recall + EMs reduces vividness and emotionality vs. recall only.	EMs affect intrusive images about the future.
Onderdonk and van den Hout, 2016	Empirical study	HC (n = 17)	EM	Visual task analogous to EM	Study 1 found that RT was slowest in the EM condition. Study 2 found decreases in memory vividness and emotionality after EM. The visual analogous task was similar to the control condition.	Performing EM taxes more WM resources and has greater impact on both memory vividness and emotionality than analogous visual tasks. This demonstrates that the effects observed in EMDR treatment are the result of more than occupying WM systems with visual stimuli alone.
Boukezzi et al., 2017b	Empirical study	HC (n = 18)	BLS coupled with positive/negative conditioning	positive/negative conditioning without BLS	Fear extinction were facilitated by BLS and associated with reduced skin conductance.	The BLS effect during fear extinction may rely on taxation of working memory, reducing vividness and emotionality, or may provoke memory reconsolidation.
Littel et al., 2017b	Empirical study	HC (*n =* 74)	EM	Recall with no EM	In the absence of arousal, neutral memory vividness did not decrease after recall + EM relative to recall only.	Results of the current study indicate that arousal is a prerequisite for the effectiveness of dual task interventions.
Patel and McDowall, 2017	Empirical study	HC (*n =* 31)	EM	Recall with no EM	Fast eye movements lowered vividness but not emotionality self-ratings ratings.	Extension to the working memory explanation. The eye movements lower the number of intrusive thoughts of negative memories during suppression.

*EM, eye movements; EMDR, eye movement desensitization and reprocessing; HC, healthy controls; NA, not applicable; OR, orienting response; PTSD, posttraumatic stress disorder; VSSP, visuospatial sketchpad WM, working memory; BLS, bilateral stimulation*.

**Table 2:** Psychophysiological models (*n* = 18).

**Author, year**	**Type of study**	**Sample (n)**	**EM/Full protocol**	**Control condition**	**Main findings**	**Conclusions**
Kuiken et al., 2002	Empirical study	HC (*n =* 25)	EM	Visual fixation (noEM)	EM facilitates attentional and semantic flexibility.	EM induced attentional and semantic flexibility facilitates OR and transformations in the clients traumatic memory.
Barrowcliff et al., 2003	Empirical study	HC (*n =* 18)	EM	Visual fixation (noEM) High-low frequency tones Attentional task	Lower levels of electrodermal arousal were identified in EM compared to noEM.	EM facilitate a process of psycho-physiological de-arousal
Barrowcliff et al., 2004	Empirical study	HC (*n =* 80)	EM	Stationary eyes (noEM)	EM resulted in decreased psychophysiological response and reductions on vividness and emotionality in positive and negative memories.	EM facilitate a process of psycho-physiological de-arousal
Aubert-Khalfa et al., 2008	Empirical study	HC = 6	EMDR	Pre-post treatment Within-group	Post-treatment reductions of clinical scores and psychophysiological response.	Successful EMDR treatment reduces psychophysiological arousal associated with trauma
Elofsson et al., 2008	Empirical study	PTSD (*n =* 13)	EMDR	NA	Psycho-physiological changes compatible with de-arousal during EMDR.	EM during EMDR activate cholinergic and inhibit sympathetic systems, similarly to the changes observed during REM sleep
Schubert et al., 2008	Empirical study	PTSD (*n =* 10)	EMDR	NA	EMDR provokes (i) an increase of psychophysiological response at stimulation onsets and (ii) stress related arousal during ongoing stimulation. Across the entire EDMR significant decreases of psycho-physiological activity was observed.	EMDR is associated with autonomic de-arousal over time
Sack et al., 2008	Empirical study	PTSD (*n =* 10)	EMDR	NA	Treatment with EMDR was followed by a significant reduction of subjective disturbance; trauma related symptoms and reduced psycho-physiological reactivity.	The successful processing of trauma mediated by repetitive ORs causes an habituation of the psycho-physiological response.
Frustaci et al., 2010	Empirical study	HC (sub-syndromal PTSD) = 4	EMDR	Pre-post treatment Within group	EMDR decreased symptoms and increased parasympathetic tone.	Results support physiological de-arousal reductions driven by EMDR also in sub-syndromal PTSD.
Kapoula et al., 2010	Empirical study	HC (*n =* 7)	EMDR	NA	EMDR decrease the number of saccade intrusions and increase the smooth components of the ocular pursuit.	EMDR reduces distress mediated by cholinergic effects known to improve ocular pursuit.
Hornsveld et al., 2010	Empirical study	HC (*n =* 60)	EM	recall + noEM; recall + music	Greater decline in emotionality and concentration after EM compared to recall-only and recall-with-music.	EM reduce vividness resulting in detachment from the trauma.
El Khoury-Malhame et al., 2011	Empirical study	HC (*n =* 19)	EMDR	Emotional Stroop Target detection task	EMDR contributes to removal of PTSD symptoms vs. control conditions. After successful EMDR therapy patients respond similarly to controls in attentional tasks.	Removal of PTSD symptoms with EMDR eliminates attentional bias towards aversive cues.
Stickgold, 2002, 2008	Speculative theory	NA	NA	NA	NA	EMDR induces a neurobiological state similar to that of the REM sleep that contributes to integrate traumatic memories into general semantic networks.
Sharpley et al., 1996a	Empirical study	HC (*n =* 20)	EM	Rolling eyes upwards	EM were not associated with increased relaxation as measured by heart rate and alpha activity.	EMDR effects does not rest upon alpha-induction or cause overall relaxation.
Schubert et al., 2011	Empirical study	HC (*n* = 64)	EMDR	EMDR with no-EM	EMDR with EM was associated with greater reduction of distress. EMDR led to greater dearousal on physiological variables.	The dual-attention tasks in EMDR create orienting responses and short-term dearousal which may aid in the processing and integration of trauma memories. The relaxation response associated with EMs in EMDR may serve to moderate arousal throughout treatment sessions.
Raboni et al., 2014	Empirical study	PTSD (*n =* 13) HC (*n* = 11)	EMDR	Pre-post treatment Within group Between group	EMDR decrease symptoms of depression and anxiety in PTSD.	Reduced sympathetic activation may explain the improvements observed after EMDR.
Farina et al., 2015	Empirical study	PTSD (*n* = 6)	EMDR	Pre-post design	EMDR was associated with alpha power increases in the left inferior temporal gyrus and HRV. Finally, the values of lagged coherence were negatively associated with subjective units of disturbance and positively associated with parasympathetic activity.	Results suggest that EMDR leads to an integration of dissociated aspects of traumatic memories and, consequently, a decrease of hyperarousal symptoms
Schubert et al., 2016	Empirical study	PTSD (*n* = 20)	EMDR	Pre-post treatment Within group	EMDR treatment was followed by significant reductions in PTSD, depression, and anxiety symptoms. Decreases in heart rate, respiration rate, and skin conductance indicated physiological dearousal within treatment sessions.	Support for the orienting response-relaxation and physiological dearousal during and after successful EMDR treatment
Pagani and Carletto, 2017	Speculative theory	NA	NA	NA	NA	Slow-wave sleep, like EM in EMDR has a key role in memory consolidation and in the reorganization of distant functional networks, as well as lead to a weakening of traumatic episodic memory and a reconsolidation of new associated information.

*EM, eye movements; EMDR, eye movement desensitization and reprocessing; HC, healthy controls; NA, not applicable; OR, orienting response; PTSD, posttraumatic stress disorder; REM, rapid eye movement*.

**Table 3:** Neurobiological models (*n* = 37).

**Author, year**	**Type of study**	**Sample (*n*)**	**EM/Full protocol**	**Control condition**	**Findings/Outcome**	**Implications for the mechanism of action**
**CHANGES IN INTERHEMISPHERIC CONNECTIVITY**						
Christman et al., 2003	Empirical study	HC (*n* = 280)	Saccadic and smooth pursuit EM	Horizontal vs. vertical EM vs. no EM	Saccadic EM enhanced episodic memory retrieval.	EM enhance interhemispheric interaction facilitating retrieval of episodic memories.
Christman et al., 2006	Empirical study	HC (*n* = 86)	Saccadic and smooth pursuit EM	Horizontal vs. vertical EM vs. no EM	Saccadic EM led to recall of earlier childhood events.	EM enhance interhemispheric interaction facilitating retrieval of episodic memories.
Rasolkhani-Kalhorn and Harper, 2006	Speculative theory	NA	NA	NA	NA	Depotentiation may be the biological basis of EMDR. Induction of low frequency stimulation by EM can lead to modification of fear memory traces.
Parker and Dagnall, 2007	Empirical study	HC (*n* = 102)	EM	Horizontal vs. vertical EM vs. no EM	Saccadic eye movements increased true recognition of words and decreased false recognition.	EM may increase interhemispheric interaction leading to increased contextual information associated with previously learnt items.
Parker et al., 2008	Empirical study	HC (*n* = 96)	EM	Horizontal vs. vertical EM vs. no EM	EM increased associative recognition and recollection.	EM (dual processing task) improve performance of associative learning tasks. This mechanisms may be explained by increased interhemispheric interaction.
Parker et al., 2009	Empirical study	HC (*n* = 72)	EM	Horizontal vs. vertical EM vs. no EM	Horizontal EM increase true memories and recollection EM also decreased the magnitude of the misinformation effect.	Horizontal EM enhance the monitoring and dual processing of source memories.
Brunyé et al., 2009	Empirical study	HC (*n* = 72)	EM	Horizontal vs. vertical EM vs. no EM	Horizontal EM increased recognition in verbal and non-verbal memory tests.	The effects of horizontal EM in EMDR may induce increased interhemispheric brain activity.
Nieuwenhuis et al., 2013	Empirical study	HC (*n* = 50)	EM	Horizontal EM no EM (on-screen fixation) simultaneous tactile stimulation simultaneous auditory stimulation	Horizontal EM and tactile stimulation enhance memory retrieval.	EM-driven bilateral stimulation of the brain increase functional connectivity between the two hemispheres, leading to enhanced memory retrieval.
Keller et al., 2016	Empirical study	HC (*n* = 30)	EM	Stationary eyes	EM were not associated with enhanced interhemispheric coherence but with were associated with intrahemispheric coherence in the right frontal and temporal areas.	A cortical coherence extension for the interhemispheric coherence hypothesis is suggested.
Yaggie et al., 2016	Empirical study	HC (*n =* 46)	EM	Stationary eyes Between/within-groups experimental design	No differences in vividness and emotional valence between all conditions. No significant increases in interhemispheric coherence measured by EEG. Increases in intrahemispheric coherence associated to EM.	Support for a two-stage cortical coherence model, integrating findings from other hypothesis and models.
**STRUCTURAL AND FUNCTIONAL BRAIN CHANGES ASSOCIATED WITH EMDR THERAPY**						
O'Driscoll et al., 1998	PET	HC = 10	EM	Saccadic vs. smooth pursuit movements	Saccadic movements are associated with increased metabolism of the frontal cortex.	Differential activation between smooth pursuit and saccadic eye movements.
Levin et al., 1999	SPECT	PTSD = 6	EMDR	Pre-post treatment Within group	Post EMDR hyper activation of ACC and left PFC.	Successful EMDR treatment in PTSD may enhance the ability to differentiate real from imagined threat.
Lamprecht et al., 2004	EEG, ERP	PTSD = 10	EMDR	Pre-post treatment Within group	Post EMDR reduced OR to novel stimuli and arousal level.	Clinical improvement of trauma in PTSD patients may be related to changes in information processing.
Lansing et al., 2005	SPECT	PTSD = 6	EMDR	Pre-post treatment Within group	Changes in perfusion post EDMR treatment. Decrease perfusion in the left and right occipital, left parietal, and right precentral lobes Increased perfusion in the left inferior frontal gyrus.	Significant functional differences in brain activity from pre- to post-EMDR imaging consistent with psychotherapy effects on depression and anxiety disorders.
Oh and Choi, 2007	SPECT	PTSD = 2	EMDR	Pre-post treatment Within group	Increased perfusion in PFC and decreased perfusion in temporal association cortex.	EMDR treatment reverse the functional imbalance between the limbic area and the prefrontal cortex.
Letizia et al., 2007	MRI	PTSD = 1	EMDR	Pre-post treatment Single case	Increased hippocampal volume.	Psychotherapy may induce alterations in gene expression and structural changes in the brain.
Pagani et al., 2007	SPECT	PTSD = 15 HC = 22	EDMR	Pre-post treatment Within group Between group	Reduction toward normalization in EMDR respondents in pre-limbic cortices and increases in the PFC.	The imaging findings are consistent with previously described imaging changes of psychotherapy on anxiety disorders.
Propper et al., 2007	EEG	HC = 22	EM	Horizontal vs. vertical EM vs. noEM	EM led to decreased interhemispheric coherence.	EM may induce changes but not necessarily decreases in interhemispheric interaction.
Harper et al., 2009	EEG	PTSD = 6	EMDR	Within group analysis	Symptoms of PTSD were reduced after EMDR. EEG activity was compatible to de-potentiation memory synapses.	Treatment gains in EMDR may result from de-potentiation of fear in memory synapses.
Ohtani et al., 2009	NIRS	PTSD = 13	EMDR	Pre-, during, post-treatment Within group	Decreased activity in PFC during recall with EM.	Reduced activity in the PFC may be part of the biological basis for the efficacy of EMDR in PTSD.
Grbesa et al., 2010	EEG	PTSD = 1	EMDR	Pre-, during and post- treatment Within subject	Low level electrocortical amplitude was observed during EMDR. Increased EEG amplitude was observed after successful treatment.	Successful EMDR treatment correlates with sudden increases of electrocortcial amplitude activity.
Nardo et al., 2010	MRI	PTSD = 21 HC = 22	EMDR	Between group	Lower GM density was found in the left posterior cingulate, parahippocampal, limbic and paralimbic cortices in non- responders to EMDR therapy.	GM lower density in limbic and paralimbic cortices is associated with PTSD diagnosis, trauma load, and EMDR treatment outcome, suggesting that PTSD is characterized by memory and dissociative disturbances.
Bossini et al., 2011	MRI	PTSD = 10	EMDR	Pre-post treatment Within group	Increased hippocampal volume post EMDR.	EMDR may induce alterations in gene expression and structural changes in the brain.
Pagani et al., 2012	EEG	PTSD = 10 HC = 10	EMDR	Pre-, during and post treatment Within and between group	Activations shifted from frontal to temporal regions over the course of the treatment.	Traumatic events are processed at cognitive level following successful EMDR therapy.
Samara et al., 2011	EEG	HC (*n* = 14)	EM	noEM	Interhemispheric phase and amplitude coherence in EEG were not affected by EM. There were no associations between changes in EM-related interhemispheric connectivity and memory performance.	These findings do not support the interhemispheric interaction hypothesis.
Landin-Romero et al., 2013	fMRI	Subsyndromal traumatized bipolar patient (*n* = 1) HC = 30	EMDR	Pre-post treatment Between and within group	Post-treatment normalization of patterns of activation and deactivation.	EMDR may modulate large scale networks in the brain
Herkt et al., 2014	fMRI	HC = 20	Alternating BLS	Non alternating BLS No stimulation	Specific increase in activation of the right amygdala for the bilateral alternating auditory stimulation. Decrease activation of the dorsolateral prefrontal cortex associated to alternating BLS.	Support for increase in limbic processing along with decreased frontal activation as the neurobiological correlate of the therapeutic reintegration of information.
Boukezzi et al., 2017a	MRI	PTSD (*n* = 18)	EMDR	Supportive therapy	EMDR was associated with grey matter increases in the prefrontal cortex.	EMDR-driven symptom removal is associated with enhancement of brain structures involved in emotional regulation.
Littel et al., 2017a	Empirical study	HC (*n* = 56)	EM	Eyes stationary	No effects of EM on memory emotionality when associated with blockage of noradrenaline.	Noradrenaline is crucial for EMDR effectiveness.
Bossini et al., 2017	MRI	PTSD (*n* = 19) HC (*n* = 19)	EMDR	Pre-post design	EMDR was associated with increased grey matter volume in thalamus and parahippocampal regions.	EMDR mechanism of action work at the level of the thalamus, an area implicated in PTSD.
Thomaes et al., 2016	fMRI	PTSD (*n* = 8)	EM	Recall with no EM	Recall with EM is associated with reduced activation in amygdala and reduced prefrontal connectivity.	EM reduce activity and connectivity in emotional processing related areas.
Laugharne et al., 2016	MRI	PTSD (*n* = 20)	EMDR	Prolonged exposure	Left amygdala mean volume increased following EMDR treatment but not exposure.	Results suggest different underlying processes for the efficacy of EMDR and prolonged exposure.
Jung et al., 2016	MRI	PTSD (*n =* 17) HC (*n* = 11)	EMDR	Pre-post design	Successful treatment showed significant effects on global and local network properties.	Subthreshold manifestation of PTSD may be due to a disruption in the optimal balance in the functional brain networks and that this disruption can be ameliorated by psychotherapy.
Pagani et al., 2015	Empirical study	noPTSD trauma (*n* = 40) HC (*n* = 20)	EMDR	Pre-post design	Orbitofrontal activity shifted to posterior associative regions post-treatment. Participants with chronic exposure to trauma showed similar cortical firing at both stages.	During EMDR memory retention of the traumatic event moves from regions with implicit emotional valence to association areas in which the experience is integrated and consolidated.
Rimini et al., 2016	Empirical study	HC (*n* = 21)	EMDR	Pre-post design	EM during EMDR were associated with increased prefrontal oxygenation during recall of aversive memories.	EM were correlated with a reduced oxy-Hb concentration, which may be linked to a reduced working activity of PFC.
Amano and Toichi, 2016b	Empirical study	HC (*n* = 15)	EMDR	Pre-post design	EM was associated with a significant increase in oxy-Hb in the right superior temporal sulcus and a decrease in the wide bilateral areas of the PFC.	EM may help the recall of pleasant memories. The reduction in the PFC suggests that EM induce relaxation.
Amano and Toichi, 2016a	Empirical study	PTSD (*n* = 7)	EMDR	Pre-post design	EMDR was associated with a significant reduction in the right temporal cortex, and a trend toward a reduction in the left orbitofrontal cortex.	Successful EMDR treatment involves brain regions related to memory representation and emotion.

*ACC, anterior cingulate cortex; EEG, electroencephalogram; EM, eye movements; EMDR, eye movement desensitization and reprocessing; ERP, event related potentials; fMRI, functional magnetic resonance imaging; HC, healthy controls; GM, gray matter; MRI, magnetic resonance imaging; NIRS, near-infrared spectroscopy; NA, not applicable; OR, orienting response; PTSD, posttraumatic stress disorder; PET, positron emission tomography; PFC, prefrontal cortex; SPECT, single photon emission computer tomography; BLS, bilateral stimulation*.

## Discussion

### Psychological models

#### Classic conditioning: orienting and relaxation responses

Dyck was the first author to provide an account of the underlying mechanism of EMDR, largely in terms of classic conditioning theory (Dyck, 1993). He argued that re-experiencing the trauma in the context of the desensitization session would operate as an extinction trial of the traumatic experience. Unfortunately, Dyck did not back up this hypothesis with empirical data. Other psychological models have attempted to explain the treatment gains of EMDR through similar learning and adaptive mechanisms, such as the orienting response (OR). Pavlov first described the orienting (or investigatory) response in 1927. The OR is a natural attentional reflex that can occur with any novel environmental stimulus and produces a specific set of changes that increase readiness to respond to danger. The OR toward any stimulus that constitute a potential threat manifests itself as an initial freeze response accompanied by changes in autonomic responses that include increased blood flow, heart rate, and skin conductance. In the absence of danger, this initial response is rapidly replaced with a feeling of relaxation. According to some authors, this relaxation response holds the potential to desensitize the traumatic memory, suppressing its associated disturbance. Armstrong and Vaughan used this idea to propose an extinction model whereby the EMs trigger an orienting response that (i) facilitates access to the traumatic memory without avoidance and (ii) causes subsequent rapid extinction after the determination of no immediate threat (Armstrong and Vaughan, 1996).

Similarly, MacCulloch and Feldman (1996) and Wilson et al. (1996) proposed a combination of Pavlovian and Darwinian theories whereby the dual attention task provoked by the EMs serves to trigger an OR. This OR pairs an adaptive explorative response with clinically induced unpleasant memories to remove their negative effect. These authors have suggested a similar role to other forms of BLS (i.e., tactile or auditory) in eliciting the OR. This initial analysis has been followed by several psychophysiological studies that have leaned support to the central role of the OR as the underlying mechanism of EMDR, using EMs only (Kuiken et al., 2002; Barrowcliff et al., 2003, 2004) and the full EMDR protocol (Aubert-Khalfa et al., 2008; Sack et al., 2008; Schubert et al., 2008; Frustaci et al., 2010), mostly in healthy individuals but also in clinical populations (Schubert et al., 2016). The results of these studies are summarized in the corresponding section for psychophysiological models.

#### The working memory account

In 1974, Baddeley and Hitch introduced the multicomponent model of working memory (Baddeley and Hitch, 1974). This theory proposes a “central executive” system responsible for the integration and coordination of information stored in different slave subsystems. One of these subsystems is the phonological loop, which stores verbal and auditory information. Another is the visuospatial sketchpad, which stores visuospatial information. According to the working memory model, during EMDR sessions, memories are held in the visuospatial sketchpad. The working memory hypothesis suggests that the dual task (i.e., the EMs and the visual imagery) draw on the limited-capacity of the visuospatial sketchpad and central executive working memory resources. The competition in resources will impair imagery, and as such, the disturbing images would become less emotional and vivid. The working memory account also argues that the degradation of a traumatic image held in working memory provides patients with a healthy sense of distance from a traumatic event.

Sharpley et al. were the first to introduce the idea that the effect of EMDR is mediated by the distancing from the traumatic memory and the reduction of imagery vividness (Sharpley et al., 1996b). Years later, researchers would demonstrate that this effect is mediated by the EMs disrupting working memory resources, thereby reducing vividness and decreasing the emotionality of traumatic imagery (Andrade et al., 1997; Kavanagh et al., 2001). Follow up studies also found a significant role of EMs in the emotional detachment from traumatic memories (Baddeley and Andrade, 2000; van den Hout et al., 2013). In support of taxing working memory resources, analog research proved that implementing other demanding tasks during recall also reduced vividness and emotionality of negative memories (Engelhard et al., 2010b; de Jongh et al., 2013). Research on the working memory hypothesis has consistently demonstrated that performance is degraded when participants engage in two simultaneous tasks that require the same working memory resources, suggesting that the EMs in EMDR impairs the ability to hold a visual image in conscious awareness, resulting in the degradation of its vividness (Andrade et al., 1997; Kavanagh et al., 2001; van den Hout et al., 2001; Gunter and Bodner, 2008; Maxfield et al., 2008). Further research have refined these results, with the finding that the EMs are superior to other forms of BLS, such as auditive “beeps” and relaxing music, in decreasing the vividness and emotionality of disturbing memories in healthy participants (Hornsveld et al., 2010, 2011; van den Hout et al., 2010, 2011a, 2012).

Other authors have proposed a different mechanism to taxing working memory in decreasing vividness and emotionality whereby the EMs would change the somatic perceptions accompanying retrieval toward relaxation, resulting in decreased affect and therefore decreased vividness of the imagery (van den Hout et al., 2001, 2013; Lilley et al., 2009). This explanation has many similarities to the reciprocal inhibition techniques (i.e., systematic desensitization) first described by Wolpe. Here, a state incompatible with the anxiety (i.e., relaxation) is evoked at the same time as the anxiety-provoking stimuli, ultimately leading to its desensitization (Wolpe, 1954).

### Psychophysiological models

#### Physiological changes associated with the orienting response

In her revision of the EMDR principles and procedures, Shapiro suggested that the EMs and the dual attentional task led to specific psychophysiological changes that may underlie treatment efficacy. A set of studies has strived to determine whether the EMs indeed produce physiological effects and to identify the nature of these changes.

Wilson et al. were first to report within-subject psychophysiological changes in participants receiving a single session of EMDR (Wilson et al., 1996). They observed that heart rate and galvanic skin response decreased over a set of EMs and that the fingertip skin temperature was significantly higher at the end of the treatment session than at the start. In addition to these effects, the EMs were accompanied by changes in respiratory patterns, consistent with a relaxation response. These physiological changes are compatible with a de-arousal response following EMDR treatment. Elofsson et al. recorded and compared several psychophysiological measurements during EMs vs. phases without EMs. They found that pulse rate went down during EMs and up again afterward, an effect that became more and more pronounced as the session proceeded. Finger temperature increased immediately after the onset of EMs and continued to increase steadily before dropping immediately when the EMs ceased. On the other hand, skin conductance and heart rate were lowered during stimulation. All these changes are compatible with an increased parasympathetic contribution to autonomic activity (Elofsson et al., 2008). Barrowcliff et al. found that skin conductance was reduced during the horizontal EMs in healthy individuals (Barrowcliff et al., 2003). Sack et al. exposed 10 patients with PTSD to standard EMDR treatment and examined effects within and between stimulation sets on different respiration and heart measurements (Sack et al., 2008). The onset of each stimulation period was instead associated with a sharp increase in parasympathetic tone. This was followed by increased respiration rate and decreased heart rate during ongoing stimulation, indicating stress-related arousal. The trend across entire sessions was one of physiological de-arousal.

### REM sleep

In her initial description of the EMD theory, Shapiro suggested that the rhythmic, multi-saccadic EMs in EMDR may work as a brain-inhibitory mechanism to reduce anxiety when associated with the traumatic memory, in the same way the material surfacing during dreaming is desensitized by rapid eye movement (REM). This apparent analogy between REM sleep and EMDR was further developed by Stickgold, who proposed the REM hypothesis for the mechanism of action of EMDR. According to this hypothesis, the EMs in EMDR would induce a similar brain state to that occurring during REM sleep. Years of sleep research that has demonstrated that REM sleep serves a number of adaptive functions, including memory consolidation via the integration of emotionally charged autobiographical memories into general semantic networks (Born et al., 2006; Stickgold and Wehrwein, 2009). Similarly, EMDR would promote the reorganization of the traumatic memories, reducing the strength of the traumatic episodic memories that are mediated by the hippocampus and the associated negative emotion processed by the amygdala (Stickgold, 2002, 2008).

This hypothesis has received some indirect support from psychophysiological research. Elofsson et al. have argued that the physiological profile of EMDR fits well with the REM account (Elofsson et al., 2008; Sondergaard and Elofsson, 2008). Indirect evidence of REM-like mechanisms mediating the therapeutic effect of EMDR has been provided in a study by Raboni et al. where improved sleep and partial recovery of depressive and anxiety symptoms was observed in 13 PTSD patients after successive treatment with EMDR (Raboni et al., 2014). The authors speculated that the improvements observed after treatment where mediated by an EMDR-driven reduction of the sympathetic activation and suggested that EMDR played a role in restoring normal sleep patterns and lowering the probability of developing PTSD after a traumatic event. Nonetheless, it should be noted that there is lack of studies addressing the REM hypothesis directly. Indeed, the smooth eye pursuit that occurs during BLS in EMDR therapy is actually very different from the saccadic movements elicited during REM sleep. Instead, recent speculative theories associate the EM in EMDR to EM during slow-wave sleep, in terms of both the smooth pursuit and frequency (Pagani and Carletto, 2017; Pagani et al., 2017). Slow-wave sleep has a key role in memory consolidation and in the reorganization of distant functional networks, and leads to weakening of traumatic memories and a reconsolidation of new information. Similarly, other authors suggest that depotentiation, induced by low frequency stimulation (i.e., smooth EM pursuit), may be the biological basis of EMDR removing fear memory traces. These theories, however, remain to be tested empirically.

### Neurobiological models

The advent of non-invasive neuroimaging techniques such as the electroencephalogram (EEG), single-positron emission computed tomography (SPECT), near-infrared spectroscopy (NIRS) and structural and functional magnetic resonance imaging (sMRI, fMRI) have enabled the *in-vivo* examination of structural and functional brain changes. Neuroimaging techniques have been used with relative success in an attempt to shed light on the neurobiological correlates of diverse psychotherapies (Linden, 2006; Abbass et al., 2014; Weingarten and Strauman, 2015). Early data from different functional and anatomical studies in PTSD have supported neurobiological models that can be used to examine changes after intervention with EMDR and other psychotherapies (Lindauer et al., 2005; Bryant et al., 2008). These findings have provided a solid foundation to direct research efforts, in order to unravel the brain correlates underlying the efficacy of EMDR.

#### Changes in interhemispheric connectivity

A set of studies in non-clinical populations have tried to explain the treatment gains of EMDR based on changing interactions between the left and right brain hemispheres. Specifically, some researchers have speculated that the EMs in EMDR facilitate associative memory processing and episodic memory retrieval through increased interhemispheric communication via the corpus callosum. This hypothesis is partially based on a previous functional imaging study that has shown that saccadic eye movements generated more frontal cortical activity than do smooth pursuit eye movements (O'Driscoll et al., 1998). The effect of different conditions of EMs (i.e., saccadic vs. smooth ocular pursuit; horizontal vs. vertical EMs) on episodic memory and interhemispheric activity has been examined in a set of studies using EEG. These studies showed that saccadic horizontal EMs enhanced memory retrieval while significantly decreasing false memories. This effect was further mediated by changes in interhemispheric interaction driven by the EMs (Christman et al., 2003, 2006; Propper et al., 2007; Brunyé et al., 2009; Nieuwenhuis et al., 2013). Other studies have found that saccadic EMs facilitate processing of associative memories, lending partial support to this hypothesis (Parker and Dagnall, 2007; Parker et al., 2008, 2009). In recent years, an extension of the interhemispheric connectivity hypothesis have been suggested, including a two-stage cortical coherence model whereby *intra*-hemispheric changes in the right hemisphere may occur along with interhemispheric changes (Keller et al., 2016; Yaggie et al., 2016).

#### Neural integration and thalamic binding model

Empirical studies of the past decade have shown the thalamus to be centrally involved in the integration of perceptual, somatosensory, memorial, and cognitive processes; a process alternatively referred to as thalamo-cortical temporal binding or neural global mapping (Llinás and Ribary, 2001; Llinas et al., 2002). The thalamo-cortical binding model serves as a theory for the integration of sensory information and it is supported by neuroimaging studies that consistently find decreases in thalamic activity in PTSD (Lanius et al., 2001, 2003). This model has been proposed to explain the effects of the EMs on the neural networks. Bergmann has suggested that the BLS facilitates the subsequent activation of the ventrolateral and central lateral thalamic nuclei via activation of the lateral cerebellum (Bergmann, 2008). Accordingly, the activation of this circuitry is hypothesized to facilitate the integration of somatosensory, memory, cognitive, emotional, and synchronized hemispheric functions that are disrupted in PTSD. It is important to note that this is just a speculative theory, as this model has not been empirically tested yet. Bergmann has proposed a range of neurobiological research designs capable of testing the role the EMs (or alternate forms of BLS) on thalamic function, interhemispheric coherence and temporal binding (Bergmann, 2012).

On a similar scope, Corrigan has proposed that auditory, visual, and tactile BLS would facilitate the simulation of thalamo-cingulate tracts (Corrigan, 2002). This stimulation would lead to the deactivation of the ventral—affective—anterior cingulate gyrus, which in turn would enable the reciprocal inhibition of the dorsal (cognitive) anterior cingulate gyrus. This cascade of brain functional changes would ultimately result in increased cognitive control over overreacting affective processing systems and to the reduction of the emotional distress. This hypothesis has the support of several years of neuroimaging research has shown that these neuronal mechanisms are altered in PTSD (Pitman et al., 2012). A number of recent functional neuroimaging studies have reported activity changes in these neuronal networks after EMDR treatment, providing further support for this hypothesis (Levin et al., 1999; Lansing et al., 2005; Landin-Romero et al., 2013) [for more details on these studies see section below].

#### Structural and functional brain changes associated with EMDR therapy

In recent years, a new wave of increasingly sophisticated neuroimaging studies has been carried out to uncover the neurobiological underpinnings of EMDR. These studies seem better suited to answer persistent questions surrounding the mechanism of action of EMDR while addressing some of the limitations of early research. In particular, studies examining neuroimaging and behavioral changes “on-line,” before, during and after therapy, hold promise to unravel the neurobiological signatures of EMDR.

A small set of brain imaging studies has investigated the structural brain correlates of EMDR therapy, with a focus on memory (e.g., Letizia et al., 2007) and emotion processing structures. Nardo et al. performed a magnetic resonance imaging [MRI] study in 21 PTSD patients compared with 22 healthy controls (Nardo et al., 2010). They found decreased gray matter density in several limbic and paralytic regions in patients who did not respond to EMDR compared to EMDR responders. Lower gray matter density in the posterior, parahippocampal and insular cortices was correlated with PTSD diagnosis, trauma load and poor therapy outcome, suggesting that reduced neuronal integrity in these regions may drive the lack of response to therapy. Bossini et al. examined structural changes in 10 patients with PTSD who had the hippocampi manually delineated using high-resolution MRI scans (Bossini et al., 2011). After 8 weeks of EMDR treatment, patients no longer met PTSD criteria and showed significant bilateral increases of hippocampal volume, which led the authors to speculate with the possibility of volumetric effects induced by psychotherapy. However, this interpretation should be taken with caution, as these structural changes might have been derived by neurogenesis or increased water/electrolyte content.

In the first functional imaging study, Levin and cols. examined changes in metabolism with single-proton emission computer tomography [SPECT] and a symptom provocation paradigm before and after three sessions of EMDR in one patient with PTSD (Levin et al., 1999). The results showed increased activity post-EMDR treatment in the anterior cingulate gyrus and the left frontal lobe. The authors concluded that activation of these areas facilitates the distinction between real threats and traumatic memories that are no longer relevant to current experience. Lansing et al. also investigated brain activation using SPECT during the recall of a traumatic event in 6 traumatized police officers before and after EMDR therapy (Lansing et al., 2005). They found significant metabolic decreases in occipital, left parietal and posterior frontal lobes and metabolic increases in the left inferior frontal gyrus after successful removal of the PTSD symptoms. These findings confirmed the impact of successful EMDR therapy in increasing prefrontal control over hyperactive limbic subsystems and provided preliminary support to neural integration models. Pagani et al. confirmed these results in a further SPECT study of 15 patients and 22 non-symptomatic controls who had suffered the same trauma (Pagani et al., 2007). A subgroup of responders to EMDR showed a significant metabolic normalization after therapy in posterior cortical regions and in the hippocampus and an increase of blood perfusion in the lateral prefrontal cortex. Oh et al. have conducted the most recent SPECT EMDR study to date in two patients suffering from psychological traffic trauma compared to 10 healthy controls. They found increased metabolism in bilateral dorsolateral prefrontal cortex and decreased metabolism in the temporal association cortex following successful EMDR therapy (Oh and Choi, 2007).

Brain functional changes concurrent to EMDR therapy have also been examined with other neuroimaging techniques different to SPECT. Ohtani et al. performed the first near-infrared spectroscopy (NIRS) study to monitor brain hemodynamic changes related EMDR treatment during memory recall. In this study, recall with EMs was associated with significant decreases in blood flow in the lateral prefrontal cortex compared to recall without EMs. Further, the concentration of oxygenated hemoglobin was correlated with clinical improvement post treatment (Ohtani et al., 2009). The authors suggested that the effectiveness of EMDR might be associated with the reduction of lateral prefrontal cortex over activation during trauma-related recall. In another pioneering fMRI study, Landin-Romero et al. examined changes in brain activity in a sub-syndromal and traumatized bipolar patient following successful EMDR therapy. The results showed that symptom recovery post-treatment was followed by a functional normalization of brain activity compared to 30 matched healthy controls (Landin-Romero et al., 2013). This normalization was particularly marked in the default mode network, a subset of brain regions that that activate during self-directed mentation and that de-activates during performance of a wide range of cognitive test. It is now widely accepted that the default mode network is dysfunctional in several severe mental disorders, including PTSD (Buckner et al., 2008). The authors speculated with large scale network modulation, specifically in the default mode network, as a potential neurobiological correlate of successful EMDR therapy.

Electroencephalogram (EEG) studies have also examined brain changes after EMDR therapy in PTSD (Lamprecht et al., 2004; Harper et al., 2009; Grbesa et al., 2010; Pagani et al., 2012). In the study by Lamprecht et al. successful treatment was accompanied with reductions of the P3a component upon auditory stimulation (Lamprecht et al., 2004). In EEG research, the P3a component has been related to the engagement of attention and the processing of novel information. This finding led the authors to conclude that the observed clinical improvement was driven by changes in information processing, presumably associated to a reduced OR to novel stimuli and reduced arousal level. EEG was also used by Pagani et al. to examine on-line neurophysiological changes in PTSD patients and healthy controls during EMDR therapy (Pagani et al., 2012). When participants were focusing on the traumatic experience and during bilateral stimulation, the EEG signals relative to 20-30 s periods of bilateral stimulation were analyzed to obtain the neurobiological responses to EMDR therapy in real-time across the whole session. Results showed different neural signatures between patients and controls. Patients showed greater activity in the orbitofrontal cortex and parahippocampal gyrus while controls showed greater activation in large areas of the frontal, temporal, and parietal lobes, especially in the right hemisphere. During the first EMDR session, while still symptomatic, patients showed significantly higher activity in orbitofrontal, prefrontal and anterior cingulate cortices. Conversely, when symptoms disappeared, upon bilateral stimulation, and trauma recall, patients showed a shift in cortical activity toward associative left temporo-occipital regions. These changes were correlated to neuropsychological scores, suggesting that traumatic events are processed at the cognitive level following successful EMDR therapy.

## Conclusions

The aims of the current manuscript are twofold: first, to provide an historical overview of the introduction and development of EMDR over the last 25 years and second, to conduct a systematic review of the mechanisms of action underlying treatment gains in EMDR therapy. Eighty-seven EMDR research studies met the inclusion criteria and were organized into 3 greater categories according to different hypotheses underlying treatment gains in EMDR; psychological, psychophysiological and neurobiological. Thirty-two papers were classified as psychological models. Of these, 27 examined the working memory hypothesis, nowadays considered one of the leading explanations for the changes associated to successful EMDR therapy. Eighteen studies examined physiological effects using different measurements of autonomic function. Finally, 37 studies were classified within the neurobiological models.

Psychological models offer a theoretical framework in which an OR elicited by BLS lead to relaxation and decreased affect associated to traumatic imagery. This hypothesis has received direct experimental support from psychophysiological studies (Wilson et al., 1996; Barrowcliff et al., 2003) suggesting that distraction is not the mechanism behind these effects. The leading psychological explanation for the EMDR treatments effects is arguably the working memory model. Research on the working memory account has demonstrated reductions in vividness of disturbing memories in healthy subjects (van den Hout et al., 2011b, 2012, 2014; van Veen et al., 2015, 2016; Onderdonk and van den Hout, 2016; van Schie et al., 2016; Leer et al., 2017). However, the psychological models, and in particular the working memory account, have also received criticism. First, most studies are performed in non-clinical populations and therefore cannot address which additional mechanisms contribute to treatment effects in PTSD. Results are often not supported by concurrent neurobiological evidence and only offer partial explanations. Research on the working memory hypothesis has also relied on conditions that do not fully match those used in the standard EMDR protocol. At least two different studies have found no significant effects on memory following EMs in healthy participants (Novo Navarro et al., 2013; van Schie et al., 2015). Further, the working memory hypothesis fails to explain some well-documented effects of EMDR. These include the state of relaxation most patients experience after a few sets of bilateral stimulation (Wilson et al., 1996; Schubert et al., 2008), the spontaneous generation of positive insight, the reports of increased recognition of accurate information, attentional flexibility (El Khoury-Malhame et al., 2011) and improved retrieval of episodic memory (Shapiro and Laliotis, 2015). Finally, most early psychological models ascribe to the EMs, and later to other forms of BLS, the underlying mechanism of action of EMDR, ignoring the potential additive effects of other components of the therapy. Here, it should be noted that dual attention does not require BLS and/or EM, as this effect can also be achieved by the addition of any other “distraction task (e.g., focusing in a point in space). Further, recent studies have also found that emotional arousal (Littel et al., 2017b) and noradrenergic transmission (Littel et al., 2017a) are prerequisites for the effectiveness of dual task interventions (i.e., EMDR or others). To conclude, from the psychological model perspective, the EMs complement traumatic memory extinction by neurobiological mechanisms that are yet to be uncovered, and that these models cannot address.

Physiological studies have found that the EMs are associated with a de-arousal response driven by increased parasympathetic relative to sympathetic changes. This might happen jointly with other physiological indicators, such as an improvement in the smooth ocular pursuit during the EMs (Kapoula et al., 2010). Another hypothesis proposed that EMDR induce a physiological state similar to REM sleep but failed to explain the effects of different types of BLS (i.e., audible tones, tactile stimulation) in the reorganization of traumatic memories. Some authors consider the OR a leading candidate for such mechanism and research models to test this hypothesis have been proposed (Stickgold, 2002, 2008). However, these hypotheses are yet to be tested directly and more research is needed to determine to what extent the physiological effects driven by EMs are associated with treatment outcome.

A series of early EEG studies found that the EMs led to changes in interhemispheric interaction, facilitating in turn retrieval of episodic memories. These effects are consistent with the theoretical framework of EMDR–the AIP model- and with patient reports of increased autobiographical memory retrieval during therapy. However, some findings have cast doubt on this hypothesis. Studies have found that vertical EMs decrease memory emotionality as effectively as horizontal movements, ruling out the vertical EM as main drivers of interhemispheric changes (Gunter and Bodner, 2008). Another EEG study did not find EEG changes following EMs and improved memory retrieval, undermining any effects of increased interhemispheric communication in treatment response (Samara et al., 2011). Therefore, evidence to date seems to conclude that enhanced interhemispheric communication is not driving the changes to traumatic recollections induced by EMs, which highlights the need for more EEG research and/or other neuroimaging techniques.

Bergmann authored an influential explanation of the EMDR clinical effects integrating findings from psychological theories and neuroscience research (Bergmann, 2008). In this theory the OR “resets” the thalamus, which in turn enhances cortical temporal binding of consciousness leading to both memory retrieval and integration in semantic networks. Similarly, Corrigan has proposed that EMDR facilitates the stimulation of thalamo-cingulate tracts which would inhibit the affective subdivision of the anterior cingulate cortex, facilitating an increase in affective filtering and a concomitant decrease in affective amplification (Corrigan, 2002). Recently, neuroimaging studies have drawn from these neurobiological models and from neuroimaging findings in clinical populations to provide a significant leap in the understanding of the neurobiological correlates of EMDR. Some of these studies have examined brain functional changes associated to EMDR “online,” that is, before, during and after the application of the standard EMDR protocol, both in patients and in healthy populations. Results have described a restoration of the cortical control over the hyper aroused subcortical limbic structures (Pagani et al., 2015; Amano and Toichi, 2016b; Laugharne et al., 2016; Rimini et al., 2016; Thomaes et al., 2016; Bossini et al., 2017). However, these brain functional changes are not specific of EMDR, and similar neuronal effects can be observed in other forms of anxiety-focused psychotherapy. Moreover, the physiological foundations of these changes are currently unknown, and therefore, these neuroimaging studies cannot explain what specific mechanisms produce treatment effects in EMDR. With few exceptions, the majority of neuroimaging studies reviewed here have significant methodological limitations, including a small sample size, lack of control conditions and inconsistent conceptualization of the parameters measured. Consequently, neuroimaging research findings should be considered promising but preliminary and conclusions concerning the EMDR neurobiological correlates speculative.

Importantly, approximately half of the studies (42/87) included in this systematic review have investigated the mechanisms underlying BLS, and more specifically the EMs, compared to different control conditions. The other half (45/87) were conducted using a more holistic approach, examining mechanisms associated to the full 8-phase EMDR protocol. The specific contribution of the EMs to EMDR therapy has been a contentious issue for several years and nowadays its exact role is still under investigation (Matzke et al., 2015). The interest surrounding the EMs is partially motivated by Shapiro herself who once described it as a crucial component of EMDR therapeutic effects. This statement has been revised posteriorly, due to the evidence suggesting a similar role for other forms of BLS. The BLS and specifically the EMs, seem to be not only the distinctive characteristic of EMDR, but also the factor accounting for the faster response in EMDR therapy compared to other psychotherapies (Nijdam et al., 2012). Research has also found the EMs provide faster effects that any other forms of BLS and a recent meta-analysis of 26 randomized controlled trials reported a moderate but significant additive effect size of the EMs to treatment gains (Lee and Cuijpers, 2013). However, whether similar effects can be achieved in EMDR therapy using other dual attention tasks (i.e., not BLS) remain to be fully established.

To conclude, this review argues that the current understanding of the mechanisms of action underlying EMDR is similar to the parable of the Blind Men and the Elephant^1^ in that there is no agreed definition of what the candidate mechanisms are (i.e., EMs, BLS, dual attention, etc.) and how these mechanisms can be measured or demonstrated. EMDR is a complex therapy with a number of underlying processes simultaneously at play. Moreover, multiple mechanisms may work to produce treatment gains in EMDR; hence, an integrative model may be necessary in order to capture its myriad effects. An example of this is the recently proposed integrative model for the neural mechanism of EMDR (Coubard, 2016), which integrates theories of EMDR, neurophysiological findings on EM, and functional brain imaging of PTSD to study attentional and/or emotional disorders, such as anxiety disorders. Other integrative proposals (e.g., Sack et al., 2008; Schubert et al., 2008) suggest that dual-attention tasks ORs and short-term dearousal enable the processing of trauma memories. Through the reciprocal inhibition (i.e., pairing a relaxation response with distressing memories), the negative appraisals weaken the avoidance trauma decreases. Here, the EM (or maybe any other dual-attention task) may reduce distress to enable processing of trauma information. Although the reviewed models, often overlapping with each other, suggest directions for future research, there is a need of advocating for conceptual clarity and consistency. Future investigations should use objective measures established by previous research and evaluate several mechanisms in the context of the full EMDR protocol, before, during, and after treatment. The neurobiological foundations of temporal binding, limbic regulation, frontal lobe activation, and reciprocal anterior cingulate cortex suppression, are sufficiently interrelated to preclude mutual exclusion and should be investigated in well-designed studies, using reliable, multidimensional neurobiological indexes. Future findings will undoubtedly shed increasing light on the interrelationship of different mechanism in the successful treatment outcomes of EMDR.

## Author contributions

All authors contributed to design of the review. RL-R and AM-A conducted literature searches and RL-R wrote the first draft of the manuscript, with supervision from BLA (primary supervisor) and MP. All authors contributed to interpretation of the literature and revisions to the manuscript and all have approved the final manuscript.

### Conflict of interest statement

RL-R, AM-A, MP, and BLA have been invited as speakers in national and international EMDR conferences.

## Footnotes

^1^In the parable of the Blind Men and the Elephant, a group of six blind men touch only one part of an elephant in order to learn what it is like. Based on their individual experience they suggest that the elephant is like a wall, spear, snake, tree, fan or rope. They then compare their experience and learn that they are in complete disagreement.

## References

[B1] Abbass A. A. Nowoweiski S. J. Bernier D. Tarzwell R. Beutel M. E. (2014). Review of psychodynamic psychotherapy neuroimaging studies. Psychother. Psychosom. 83, 142–147. 10.1159/000358841 24732748

[B2] Amano T. Toichi M. (2016a). Possible neural mechanisms of psychotherapy for trauma-related symptoms: cerebral responses to the neuropsychological treatment of post-traumatic stress disorder model individuals. Sci. Rep. 6:34610. 10.1038/srep34610 27698453 PMC5048146

[B3] Amano T. Toichi M. (2016b). The role of alternating bilateral stimulation in establishing positive cognition in emdr therapy: a multi-channel near-infrared spectroscopy study. PLoS ONE 11:e0162735. 10.1371/journal.pone.0162735 27732592 PMC5061320

[B4] Andrade J. Kavanagh D. Baddeley A. (1997). Eye-movements and visual imagery: a working memory approach to the treatment of post-traumatic stress disorder. Br. J. Clin. Psychol. 36, 209–223. 10.1111/j.2044-8260.1997.tb01408.x 9167862

[B5] Armstrong M. S. Vaughan K. (1996). An orienting response model of eye movement desensitization. J. Behav. Ther. Exp. Psychiatry 27, 21–32. 10.1016/0005-7916(95)00056-9 8814518

[B6] Aubert-Khalfa S. Roques J. Blin O. (2008). Evidence of a decrease in heart rate and skin conductance responses in PTSD patients after a single EMDR session. J. EMDR Pract. Res. 2, 51–56. 10.1891/1933-3196.2.1.51

[B7] Baddeley A. D. Andrade J. (2000). Working memory and the vividness of imagery. J. Exp. Psychol. Gen. 129, 126–145. 10.1037/0096-3445.129.1.126 10756490

[B8] Baddeley A. D. Hitch G. (1974). Working Memory, in the Psychology of Learning and Motivation: Advances in Research and Theory, ed G. H. Bower New York, NY: Academic Press, 47–89.

[B9] Barrowcliff A. L. Gray N. S. MacCulloch S. Freeman T. C. MacCulloch M. J. (2003). Horizontal rhythmical eye movements consistently diminish the arousal provoked by auditory stimuli. Br. J. Clin. Psychol. 42, 289–302. 10.1348/01446650360703393 14565894

[B10] Barrowcliff A. L. Nicola S. G. Tom C. A. Malcolm J. (2004). Eye-movements reduce the vividness, emotional valence and electrodermal arousal associated with negative autobiographical memories. J. Forensic Psychiatry Psychol. 15, 325–345. 10.1080/14789940410001673042

[B11] Bergmann U. (2008). The neurobiology of EMDR: exploring the thalamus and neural integration. J. EMDR Pract. Res. 2, 300–314. 10.1891/1933-3196.2.4.300

[B12] Bergmann U. (2012). Neurobiological Foundations for Emdr Practice. New York, NY: Springer Publishing.

[B13] Bisson J. I. Ehlers A. Matthews R. Pilling S. Richards D. Turner S. (2007). Psychological treatments for chronic post-traumatic stress disorder. Systematic review and meta-analysis. Br. J. Psychiatry 190, 97–104. 10.1192/bjp.bp.106.021402 17267924

[B14] Bisson J. I. Roberts N. P. Andrew M. Cooper R. Lewis C. (2013). Psychological therapies for chronic post-traumatic stress disorder (PTSD) in adults. Cochrane Database Syst. Rev. CD003388. 10.1002/14651858.CD003388.pub4 24338345 PMC6991463

[B17] Born J. Rasch B. Gais S. (2006). Sleep to remember. Neuroscientist 12, 410–424. 10.1177/1073858406292647 16957003

[B15] Born J. Rasch B. Gais S. (2005). National Institute for Clinical Excellence. London: NICE Guidelines.

[B16] Born J. Rasch B. Gais S. (2013). Guidelines for the Management of Conditions Specifically Related to Stress. Geneva: World Health Organization. 24049868

[B19] Bossini L. Santarnecchi E. Casolaro I. Koukouna D. Caterini C. Cecchini F.. (2017). Morphovolumetric changes after EMDR treatment in drug-naive PTSD patients. Riv. Psichiatr. 52, 24–31. 10.1708/2631.27051 28287194

[B18] Bossini L. Tavanti M. Calossi S. Polizzotto N. R. Vatti G. Marino D.. (2011). EMDR treatment for posttraumatic stress disorder, with focus on hippocampal volumes: a pilot study. J. Neuropsychiatry Clin. Neurosci. 23, E1–E2. 10.1176/jnp.23.2.jnpe1 21677204

[B20] Boukezzi S. El Khoury-Malhame M. Auzias G. Reynaud E. Rousseau P. F. Richard E.. (2017a). Grey matter density changes of structures involved in posttraumatic stress disorder (PTSD) after recovery following eye movement desensitization and reprocessing (EMDR) therapy. Psychiatry Res. 266, 146–152. 10.1016/j.pscychresns.2017.06.009 28667881

[B21] Boukezzi S. Silva C. Nazarian B. Rousseau P. F. Guedj E. Valenzuela-Moguillansky C.. (2017b). Bilateral alternating auditory stimulations facilitate fear extinction and retrieval. Front. Psychol. 8:990. 10.3389/fpsyg.2017.00990 28659851 PMC5470101

[B22] Bradley R. Greene J. Russ E. Dutra L. Westen D. (2005). A multidimensional meta-analysis of psychotherapy for PTSD. Am. J. Psychiatry 162, 214–227. 10.1176/appi.ajp.162.2.214 15677582

[B23] Brunyé T. T. Mahoney C. R. Augustyn J. S. Taylor H. A. (2009). Horizontal saccadic eye movements enhance the retrieval of landmark shape and location information. Brain Cogn. 70, 279–288. 10.1016/j.bandc.2009.03.003 19346050

[B24] Bryant R. A. Felmingham K. Kemp A. Das P. Hughes G. Peduto A.. (2008). Amygdala and ventral anterior cingulate activation predicts treatment response to cognitive behaviour therapy for post-traumatic stress disorder. Psychol. Med. 38, 555–561. 10.1017/S0033291707002231 18005496

[B25] Buckner R. L. Andrews-Hanna J. R. Schacter D. L. (2008). The brain's default network: anatomy, function, and relevance to disease. Ann. N. Y. Acad. Sci. 1124, 1–38. 10.1196/annals.1440.011 18400922

[B26] Cahill S. P. Carrigan M. H. Frueh B. C. (1999). Does EMDR work? and if so, why?: a critical review of controlled outcome and dismantling research. J. Anxiety Disord. 13, 5–33. 10.1016/S0887-6185(98)00039-5 10225499

[B27] Chen Y. R. Hung K. W. Tsai J. C. Chu H. Chung M. H. Chen S. R.. (2014). Efficacy of eye-movement desensitization and reprocessing for patients with posttraumatic-stress disorder: a meta-analysis of randomized controlled trials. PLoS ONE 9:e103676. 10.1371/journal.pone.0103676 25101684 PMC4125321

[B28] Christman S. D. Garvey K. J. Propper R. E. Phaneuf K. A. (2003). Bilateral eye movements enhance the retrieval of episodic memories. Neuropsychology 17, 221–229. 10.1037/0894-4105.17.2.221 12803427

[B29] Christman S. D. Propper R. E. Brown T. J. (2006). Increased interhemispheric interaction is associated with earlier offset of childhood amnesia. Neuropsychology 20, 336–345. 10.1037/0894-4105.20.3.336 16719626

[B30] Corrigan F. (2002). Mindfulness, dissociation, EMDR, and the anterior cingulate cortex: a hypothesis. Contemp. Hypn. 19, 8–17. 10.1002/ch.235

[B31] Coubard O. A. (2016). An integrative model for the neural mechanism of eye movement desensitization and reprocessing (EMDR). Front. Behav. Neurosci. 10:52. 10.3389/fnbeh.2016.00052 27092064 PMC4820440

[B32] Davidson P. R. Parker K. C. (2001). Eye movement desensitization and reprocessing (EMDR): a meta-analysis. J. Consult. Clin. Psychol. 69, 305–316. 10.1037/0022-006X.69.2.305 11393607

[B33] de Jongh A. Ernst R. Marques L. Hornsveld H. (2013). The impact of eye movements and tones on disturbing memories involving PTSD and other mental disorders. J. Behav. Ther. Exp. Psychiatry 44, 477–483. 10.1016/j.jbtep.2013.07.002 23892070

[B34] Doering S. Ohlmeier M. C. de Jongh A. Hofmann A. Bisping V. (2013). Efficacy of a trauma-focused treatment approach for dental phobia: a randomized clinical trial. Eur. J. Oral Sci. 121, 584–593. 10.1111/eos.12090 24206075

[B35] Dyck M. J. (1993). A proposal for a conditioning model of eye movement desensitization treatment for posttraumatic stress disorder. J. Behav. Ther. Exp. Psychiatry 24, 201–210. 10.1016/0005-7916(93)90022-O 7910613

[B36] Ecker B. Hulley L. Ticic L (2012), Unlocking the Emotional Brain Eliminating Symptoms at Their Roots Using Memory Reconsolidation. New York, NY:Routledge.

[B37] El Khoury-Malhame M. Lanteaume L. Beetz E. M. Roques J. Reynaud E. Samuelian J. C.. (2011). Attentional bias in post-traumatic stress disorder diminishes after symptom amelioration. Behav. Res. Ther. 49, 796–801. 10.1016/j.brat.2011.08.006 21924404

[B38] Elofsson U. O. von Schèele B. Theorell T. Söndergaard H. P. (2008). Physiological correlates of eye movement desensitization and reprocessing. J. Anxiety Disord. 22, 622–634. 10.1016/j.janxdis.2007.05.012 17604948

[B39] Engelhard I. M. van den Hout M. A. Dek E. C. Giele C. L. van der Wielen J. W. Reijnen M. J. (2011). Reducing vividness and emotional intensity of recurrent “flashforwards” by taxing working memory: an analogue study. J. Anxiety Disord. 25, 599–603. 10.1016/j.janxdis.2011.01.009 21376527

[B40] Engelhard I. M. van den Hout M. A. Janssen W. C. van der Beek J. (2010a). Eye movements reduce vividness and emotionality of “flashforwards”. Behav. Res. Ther. 48, 442–447. 10.1016/j.brat.2010.01.003 20129601

[B41] Engelhard I. M. van Uijen S. L. van den Hout M. A. (2010b). The impact of taxing working memory on negative and positive memories. Eur. J. Psychotraumatol. 1:5623. 10.3402/ejpt.v1i0.5623 22893797 PMC3402003

[B42] Faretta E. (2012). [EMDR and cognitive-behavioural therapy in the treatment of panic disorder: a comparison]. Riv. Psichiatr. 47, 19–25. 10.1708/1071.11735 22622275

[B43] Farina B. Imperatori C. Quintiliani M. I. Castelli Gattinara P. Onofri A. Lepore M.. (2015). Neurophysiological correlates of eye movement desensitization and reprocessing sessions: preliminary evidence for traumatic memories integration. Clin. Physiol. Funct. Imaging 35, 460–468. 10.1111/cpf.12184 25123377

[B44] Frustaci A. Lanza G. A. Fernandez I. di Giannantonio M. Pozzi G. (2010). Changes in psychological symptoms and heart rate variability during EMDR treatment: a case series of subthreshold PTSD. J. EMDR Pract. Res. 4, 3–11. 10.1891/1933-3196.4.1.3

[B45] Grbesa G. Simonovic M. Jankovic D. (2010). Electrophysiological changes during EMDR treatment in pateints with combat-related PTSD. Ann. Gen. Psychiatry 9:S209 10.1186/1744-859X-9-S1-S209

[B47] Gunter R. Bodner G. (2009). EMDR works but how? recent progress in the search for treatment mechanisms. J. EMDR Pract. Res. 3, 161–168. 10.1891/1933-3196.3.3.161

[B46] Gunter R. W. Bodner G. E. (2008). How eye movements affect unpleasant memories: support for a working-memory account. Behav. Res. Ther. 46, 913–931. 10.1016/j.brat.2008.04.006 18565493

[B48] Harper M. L. Rasolkhani-Kalhorn T. Drozd J. F. (2009). On the neural basis of EMDR therapy: insights from qeeg studies. Traumatology (Tallahass. Fla) 15, 81–95. 10.1177/153476560933849822973420

[B49] Hase M. Balmaceda U. M. Hase A. Lehnung M. Tumani V. Huchzermeier C.. (2015). Eye movement desensitization and reprocessing (EMDR) therapy in the treatment of depression: a matched pairs study in an inpatient setting. Brain Behav. 5:e00342. 10.1002/brb3.342 26085967 PMC4467776

[B50] Herbert J. D. Lilienfeld S. O. Lohr J. M. Montgomery R. W. O'Donohue W. T. Rosen G. M.. (2000). Science and pseudoscience in the development of eye movement desensitization and reprocessing: implications for clinical psychology. Clin. Psychol. Rev. 20, 945–971. 10.1016/S0272-7358(99)00017-3 11098395

[B51] Herkt D. Tumani V. Grön G. Kammer T. Hofmann A. Abler B. (2014). Facilitating access to emotions: neural signature of EMDR stimulation. PLoS ONE 9:e106350. 10.1371/journal.pone.0106350 25165974 PMC4148424

[B52] Hornsveld H. K. Jan H. H. Max V. Marcel A. H. (2011). Evaluating the effect of eye movements on positive memories such as those used in resource development and installation. J. EMDR Pract. Res. 5, 146–155. 10.1891/1933-3196.5.4.146

[B53] Hornsveld H. K. Landwehr F. Stein W. Stomp P. H. Smeets A. M. (2010). Emotionality of loss-related memories is reduced after recall plus eye movements but not after recall plus music or recall only. J. EMDR Pract. Res. 106–112. 10.1891/1933-3196.4.3.106

[B54] Jeffries F. W. Davis P. (2013). What is the role of eye movements in eye movement desensitization and reprocessing (EMDR) for post-traumatic stress disorder (PTSD)? a review. Behav. Cogn. Psychother. 41, 290–300. 10.1017/S1352465812000793 23102050

[B55] Jung W. H. Chang K. J. Kim N. H. (2016). Disrupted topological organization in the whole-brain functional network of trauma-exposed firefighters: a preliminary study. Psychiatry Res. 250, 15–23. 10.1016/j.pscychresns.2016.03.003 27107156

[B56] Kapoula Z. Yang Q. Bonnet A. Bourtoire P. Sandretto J. (2010). EMDR effects on pursuit eye movements. PLoS ONE 5:e10762. 10.1371/journal.pone.0010762 20505828 PMC2874012

[B57] Kavanagh D. J. Freese S. Andrade J. May J. (2001). Effects of visuospatial tasks on desensitization to emotive memories. Br. J. Clin. Psychol. 40, 267–280. 10.1348/014466501163689 11593955

[B58] Keller B. Stevens L. Lui C. Murray J. Yaggie M. (2016). The effects of bilateral eye movements on EEG coherence when recalling a pleasant memory. J. Emdr Pract. Res. 8, 113–128. 10.1891/1933-3196.8.3.113

[B59] Kristjánsdóttir K. Lee C. M. (2011). A comparison of visual versus auditory concurrent tasks on reducing the distress and vividness of aversive autobiographical memories. J. EMDR Pract. Res. 34–41. 10.1891/1933-3196.5.2.34

[B60] Kuiken D. Chudleigh M. Racher D. (2010). bilateral eye movements, attentional flexibility and metaphor comprehension: the substrate of rem dreaming? Dreaming 20, 227–247. 10.1037/a0020841

[B61] Kuiken D. Michael B. David M. Laurie S. (2002). Eye movement desensitization reprocessing facilitates attentional orienting. Imagin. Cogn. Pers. 21, 3–20. 10.2190/L8JX-PGLC-B72R-KD7X

[B62] Lamprecht F. Köhnke C. Lempa W. Sack M. Matzke M. Münte T. F. (2004). Event-related potentials and EMDR treatment of post-traumatic stress disorder. Neurosci. Res. 49, 267–272. 10.1016/j.neures.2004.02.013 15140569

[B63] Landin-Romero R. Novo P. Vicens V. McKenna P. J. Santed A. Pomarol-Clotet E.. (2013). EMDR therapy modulates the default mode network in a subsyndromal, traumatized bipolar patient. Neuropsychobiology 67, 181–184. 10.1159/000346654 23548794

[B64] Lanius R. A. Williamson P. C. Densmore M. Boksman K. Gupta M. A. Neufeld R. W.. (2001). Neural correlates of traumatic memories in posttraumatic stress disorder: a functional MRI investigation. Am. J. Psychiatry 158, 1920–1922. 10.1176/appi.ajp.158.11.1920 11691703

[B65] Lanius R. A. Williamson P. C. Hopper J. Densmore M. Boksman K. Gupta M. A.. (2003). Recall of emotional states in posttraumatic stress disorder: an fMRI investigation. Biol. Psychiatry 53, 204–210. 10.1016/S0006-3223(02)01466-X 12559652

[B66] Lansing K. Amen D. G. Hanks C. Rudy L. (2005). High-resolution brain SPECT imaging and eye movement desensitization and reprocessing in police officers with PTSD. J. Neuropsychiatry Clin. Neurosci. 17, 526–532. 10.1176/jnp.17.4.526 16387993

[B67] Laugharne J. Kullack C. Lee C. W. McGuire T. Brockman S. Drummond P. D.. (2016). Amygdala volumetric change following psychotherapy for posttraumatic stress disorder. J. Neuropsychiatry Clin. Neurosci. 28, 312–318. 10.1176/appi.neuropsych.16010006 27255857

[B68] Lee C. W. Cuijpers P. (2013). A meta-analysis of the contribution of eye movements in processing emotional memories. J. Behav. Ther. Exp. Psychiatry 44, 231–239. 10.1016/j.jbtep.2012.11.001 23266601

[B69] Lee C. W. Drummond P. D. (2008). Effects of eye movement versus therapist instructions on the processing of distressing memories. J. Anxiety Disord. 22, 801–808. 10.1016/j.janxdis.2007.08.007 17890048

[B70] Lee C. W. Taylor G. Drummond P. D. (2006). The active ingredient in EMDR: is it traditional exposure or dual focus of attention? Clin. Psychol. Psychother. 13, 97–107.

[B72] Leer A. Engelhard I. M. Lenaert B. Struyf D. Vervliet B. Hermans D. (2017). Eye movement during recall reduces objective memory performance: an extended replication. Behav. Res. Ther. 92, 94–105. 10.1016/j.brat.2017.03.002 28315585

[B71] Leer A. Engelhard I. M. van den Hout M. A. (2014). How eye movements in EMDR work: changes in memory vividness and emotionality. J. Behav. Ther. Exp. Psychiatry 45, 396–401. 10.1016/j.jbtep.2014.04.004 24814304

[B73] Letizia B. Andrea F. Paolo C. (2007). Neuroanatomical changes after eye movement desensitization and reprocessing (EMDR) treatment in posttraumatic stress disorder. J. Neuropsychiatry Clin. Neurosci. 19, 475–476. 10.1176/jnp.2007.19.4.475 18070859

[B74] Levin P. Lazrove S. van der Kolk B. (1999). What psychological testing and neuroimaging tell us about the treatment of posttraumatic stress disorder by eye movement desensitization and reprocessing. J Anxiety Disord. 13, 159–172. 10.1016/S0887-6185(98)00045-0 10225506

[B75] Lilley S. A. Andrade J. Turpin G. Sabin-Farrell R. Holmes E. A. (2009). Visuospatial working memory interference with recollections of trauma. Br. J. Clin. Psychol. 48, 309–321. 10.1348/014466508X398943 19187579

[B76] Lindauer R. J. Vlieger E. J. Jalink M. Olff M. Carlier I. V. Majoie C. B.. (2005). Effects of psychotherapy on hippocampal volume in out-patients with post-traumatic stress disorder: a MRI investigation. Psychol. Med. 35, 1421–1431. 10.1017/S0033291705005246 16164766

[B77] Linden D. E. (2006). How psychotherapy changes the brain–the contribution of functional neuroimaging. Mol. Psychiatry 11, 528–538. 10.1038/sj.mp.4001816 16520823

[B78] Littel M. Kenemans J. L. Baas J. M. P. Logemann H. N. A. Rijken N. Remijn M.. (2017a). The effects of beta-adrenergic blockade on the degrading effects of eye movements on negative autobiographical memories. Biol. Psychiatry 82, 587–593. 10.1016/j.biopsych.2017.03.012 28456330

[B79] Littel M. Malou R. Angelica M. T. Iris M. E. Marcel A. (2017b). Stress enhances the memory-degrading effects of eye movements on emotionally neutral memories. Clin. Psychol. Sci. 5, 316–324. 10.1177/216770261668729222973420

[B81] Llinás R. Ribary U. (2001). Consciousness and the brain. The thalamocortical dialogue in health and disease. Ann. N. Y. Acad. Sci. 929, 166–175. 10.1111/j.1749-6632.2001.tb05715.x 11349424

[B80] Llinas R. R. Leznik E. Urbano F. J. (2002). Temporal binding via cortical coincidence detection of specific and nonspecific thalamocortical inputs: a voltage-dependent dye-imaging study in mouse brain slices. Proc. Natl. Acad. Sci. U.S.A. 99, 449–454. 10.1073/pnas.012604899 11773628 PMC117580

[B82] Lohr J. M. DeMaio C. McGlynn F. D. (2003). Specific and nonspecific treatment factors in the experimental analysis of behavioral treatment efficacy. Behav. Modif. 27, 322–368. 10.1177/0145445503027003005 12841588

[B83] Lytle R. A. Hazlett-Stevens H. Borkovec T. D. (2002). Efficacy of Eye Movement Desensitization in the treatment of cognitive intrusions related to a past stressful event. J. Anxiety Disord. 16, 273–288. 10.1016/S0887-6185(02)00099-3 12214813

[B84] MacCulloch M. J. Feldman P. (1996). Eye movement desensitisation treatment utilises the positive visceral element of the investigatory reflex to inhibit the memories of post-traumatic stress disorder: a theoretical analysis. Br. J. Psychiatry 169, 571–579. 10.1192/bjp.169.5.571 8932885

[B85] Matzke D. Nieuwenhuis S. van Rijn H. Slagter H. A. van der Molen M. W. Wagenmakers E. J. (2015). The effect of horizontal eye movements on free recall: a preregistered adversarial collaboration. J. Exp. Psychol. Gen.. 144, e1–e15. 10.1037/xge0000038 25621378

[B86] Maxfield L. Melnyk W. T. Gordon Hayman C. A. (2008). A working memory explanation for the effects of eye movements in EMDR. J. EMDR Pract. Res. 2, 247–261. 10.1891/1933-3196.2.4.247

[B87] McGuire T. M. Lee C. W. Drummond P. D. (2014). Potential of eye movement desensitization and reprocessing therapy in the treatment of post-traumatic stress disorder. Psychol. Res. Behav. Manag. 7, 273–283. 10.2147/PRBM.S52268 25302027 PMC4189702

[B88] Moher D. Liberati A. Tetzlaff J. Altman D. G. PRISMA Group. (2009). Preferred reporting items for systematic reviews and meta-analyses: the PRISMA statement. Open Med. 3, e123–e130. 10.1371/journal.pmed.1000097 21603045 PMC3090117

[B89] Moreno-Alcázar A. Radua J. Landín-Romero R. Blanco L. Madre M. Reinares M. (2015). The EMDR therapy protocol for bipolar disorder, in Eye Movement Desensitization and Reprocessing (Emdr) Therapy Scripted Protocols and Summary Sheets: Treating Anxiety, Obsessive-Compulsive, and Mood-Related Conditions, ed Luber M. (New York, NY: Springer Publishing Co).

[B90] Nardo D. Högberg G. Looi J. C. Larsson S. Hällström T. Pagani M. (2010). Gray matter density in limbic and paralimbic cortices is associated with trauma load and EMDR outcome in PTSD patients. J. Psychiatr. Res. 44, 477–485. 10.1016/j.jpsychires.2009.10.014 19942229

[B91] Nazari H. Momeni N. Jariani M. Tarrahi M. J. (2011). Comparison of eye movement desensitization and reprocessing with citalopram in treatment of obsessive-compulsive disorder. Int. J. Psychiatry Clin. Pract. 15, 270–274. 10.3109/13651501.2011.590210 22122001

[B92] Nieuwenhuis S. Elzinga B. M. Ras P. H. Berends F. Duijs P. Samara Z. Slagter H. A.. (2013). Bilateral saccadic eye movements and tactile stimulation, but not auditory stimulation, enhance memory retrieval. Brain Cogn. 81, 52–56. 10.1016/j.bandc.2012.10.003 23174428

[B93] Nijdam M. J. Gersons B. P. Reitsma J. B. de Jongh A. Olff M. (2012). Brief eclectic psychotherapy v. eye movement desensitisation and reprocessing therapy for post-traumatic stress disorder: randomised controlled trial. Br. J. Psychiatry 200, 224–231. 10.1192/bjp.bp.111.099234 22322458

[B94] Novo Navarro P. Landin-Romero R. Guardiola-Wanden-Berghe R. Moreno-Alcázar A. Valiente-Gómez A. Lupo W.. (2016). *25 years of eye movement desensitization and reprocessing (EMDR): the EMDR therapy* protocol, hypotheses of its mechanism of action and a systematic review of its efficacy in the treatment of post-traumatic stress disorder. Rev. Psiquiatr. Salud Ment. 11, 101–114. 10.1016/j.rpsm.2015.12.002 26877093

[B95] Novo Navarro P. Marini A. M. Scott J. Landin-Romero R. Amann B. L. (2013). No effects of eye movements on the encoding of the visuospatial sketchpad and the phonological loop in healthy participants: Possible implications for eye movement desensitization and reprocessing therapy. Pers. Individ. Dif. 55, 983–988. 10.1016/j.paid.2013.08.005

[B96] Novo P. Landin-Romero R. Radua J. Vicens V. Fernandez I. Garcia F.. (2014). Eye movement desensitization and reprocessing therapy in subsyndromal bipolar patients with a history of traumatic events: a randomized, controlled pilot-study. Psychiatry Res. 219, 122–128. 10.1016/j.psychres.2014.05.012 24880581

[B97] O'Driscoll G. A. Strakowski S. M. Alpert N. M. Matthysse S. W. Rauch S. L. Levy D. L.. (1998). Differences in cerebral activation during smooth pursuit and saccadic eye movements using positron-emission tomography. Biol. Psychiatry 44, 685–689. 10.1016/S0006-3223(98)00047-X 9798071

[B98] Oh D. Choi J. (2007). Changes in the regional cerebral perfusion after eye movement desensitization and reprocessing: a SPECT study of two cases. J. EMDR Pract. Res. 1, 24–30. 10.1891/1933-3196.1.1.24

[B99] Ohtani T. Matsuo K. Kasai K. Kato T. Kato N. (2009). Hemodynamic responses of eye movement desensitization and reprocessing in posttraumatic stress disorder. Neurosci. Res. 65, 375–383. 10.1016/j.neures.2009.08.014 19729044

[B100] Onderdonk S. W. van den Hout M. A. (2016). Comparisons of eye movements and matched changing visual input. J. Behav. Ther. Exp. Psychiatry 53, 34–40. 10.1016/j.jbtep.2015.10.010 27664819

[B101] Pagani M. Amann B. L. Landin-Romero R. Carletto S. (2017). Eye movement desensitization and reprocessing and slow wave sleep: a putative mechanism of action. Front. Psychol. 8:1935. 10.3389/fpsyg.2017.01935 29163309 PMC5681964

[B102] Pagani M. Carletto S. (2017). A hypothetical mechanism of action of emdr: the role of slow wave sleep. Clin. Neuropsychiatry 14, 301–305.

[B103] Pagani M. Di Lorenzo G. Monaco L. Daverio A. Giannoudas I. La Porta P.. (2015). Neurobiological response to EMDR therapy in clients with different psychological traumas. Front. Psychol. 6:1614. 10.3389/fpsyg.2015.01614 26579006 PMC4621396

[B104] Pagani M. Di Lorenzo G. Verardo A. R. Nicolais G. Monaco L. Lauretti G.. (2012). Neurobiological correlates of EMDR monitoring - an EEG study. PLoS ONE 7:e45753. 10.1371/journal.pone.0045753 23049852 PMC3458957

[B105] Pagani M. Högberg G. Fernandez I. Siracusano Alberto (2013). Correlates of EMDR therapy in functional and structural neuroimaging – a critical summary of recent findings. J. EMDR Pract. Res. 39–38. 10.1891/1933-3196.7.1.29

[B106] Pagani M. Högberg G. Salmaso D. Nardo D. Sundin O. Jonsson C.. (2007). Effects of EMDR psychotherapy on 99mTc-HMPAO distribution in occupation-related post-traumatic stress disorder. Nucl. Med. Commun. 28, 757–765. 10.1097/MNM.0b013e3282742035 17728604

[B108] Parker A. Buckley S. Dagnall N. (2009). Reduced misinformation effects following saccadic bilateral eye movements. Brain Cogn. 69, 89–97. 10.1016/j.bandc.2008.05.009 18635303

[B107] Parker A. Dagnall N. (2007). Effects of bilateral eye movements on gist based false recognition in the DRM paradigm. Brain Cogn. 63, 221–225. 10.1016/j.bandc.2006.08.005 17027132

[B109] Parker A. Relph S. Dagnall N. (2008). Effects of bilateral eye movements on the retrieval of item, associative, and contextual information. Neuropsychology 22, 136–145. 10.1037/0894-4105.22.1.136 18211163

[B110] Patel G. J. McDowall J. (2017). The role of eye movements in EMDR: conducting eye movements while concentrating on negative autobiographical memories results in fewer intrusions. J. Emdr Pract. Res. 11, E15–E26. 10.1891/1933-3196.10.1.13

[B111] Perez-Dandieu B. Tapia G. (2014). Treating trauma in addiction with EMDR: a pilot study. J. Psychoactive Drugs 46, 303–309. 10.1080/02791072.2014.921744 25188700

[B112] Pitman R. K. Rasmusson A. M. Koenen K. C. Shin L. M. Orr S. P. Gilbertson M. W.. (2012). Biological studies of post-traumatic stress disorder. Nat. Rev. Neurosci. 13, 769–787. 10.1038/nrn3339 23047775 PMC4951157

[B113] Propper R. E. Pierce J. Geisler M. W. Christman S. D. Bellorado N. (2007). Effect of bilateral eye movements on frontal interhemispheric gamma EEG coherence: implications for EMDR therapy. J. Nerv. Ment. Dis. 195, 785–788. 10.1097/NMD.0b013e318142cf73 17984782

[B114] Raboni M. R. Alonso F. F. Tufik S. Suchecki D. (2014). Improvement of mood and sleep alterations in posttraumatic stress disorder patients by eye movement desensitization and reprocessing. Front. Behav. Neurosci. 8:209. 10.3389/fnbeh.2014.00209 24959123 PMC4050739

[B115] Rasolkhani-Kalhorn T. Harper M. L. (2006). EMDR and low frequency stimulation of the brain. Traumatology (Tallahass. Fla) 12, 9–24. 10.1177/15347656060120010222973420

[B116] Rimini D. Molinari F. Liboni W. Balbo M. Darò R. Viotti E.. (2016). Effect of ocular movements during eye movement desensitization and reprocessing (EMDR) therapy: a near-infrared spectroscopy study. PLoS ONE 11:e0164379. 10.1371/journal.pone.0164379 27783688 PMC5081184

[B117] Rogers S. Silver S. M. (2002). Is EMDR an exposure therapy? a review of trauma protocols. J. Clin. Psychol. 58, 43–59. 10.1002/jclp.1128 11748596

[B118] Sack M. Lempa W. Steinmetz A. Lamprecht F. Hofmann A. (2008). Alterations in autonomic tone during trauma exposure using eye movement desensitization and reprocessing (EMDR)–results of a preliminary investigation. J. Anxiety Disord. 22, 1264–1271. 10.1016/j.janxdis.2008.01.007 18314305

[B119] Samara Z. Elzinga B.M. Slagter H.A. Nieuwenhuis S. (2011). Do horizontal saccadic eye movements increase interhemispheric coherence? investigation of a hypothesized neural mechanism underlying EMDR. Front. Psychiatry 2:4. 10.3389/fpsyt.2011.00004 21556274 PMC3089996

[B120] Schubert S. J. Lee C. W. Drummond P. D. (2008). Psychophysiological changes during EMDR and treatment outcome. J. EMDR Pract. Res. 2, 239–246. 10.1891/1933-3196.2.4.239

[B121] Schubert S. J. Lee C. W. Drummond P. D. (2011). The efficacy and psychophysiological correlates of dual-attention tasks in eye movement desensitization and reprocessing (EMDR). J. Anxiety Disord. 25, 1–11. 10.1016/j.janxdis.2010.06.024 20709492

[B122] Schubert S. J. Lee C. W. Drummond P. D. (2016). Eye movements matter, but why? psychophysiological correlates of EMDR therapy to treat trauma in timor-leste. J. Emdr Pract. Res. 10, 70–81. 10.1891/1933-3196.10.2.70

[B123] Shapiro F. (1989a). Efficacy of the eye movement desensitization procedure in the treatment of traumatic memories. J. Trauma. Stress 2, 199–223. 10.1002/jts.249002020725855820

[B124] Shapiro F. (1989b). Eye movement desensitization: a new treatment for post-traumatic stress disorder. J. Behav. Ther. Exp. Psychiatry 20, 211–217. 10.1016/0005-7916(89)90025-6 2576656

[B125] Shapiro F. (1994). Eye Movement Desensitization and Reprocessing: Basic Principles, Protocols and Procedures. New York, NY: Guilford Press.

[B126] Shapiro F. (2001). Eye Movement Desensitization and Reprocessing: Basic Principles, Protocols and Procedures, 2nd Edn. New York, NY: Guilford Press.

[B127] Shapiro F. (2007). EMDR, adaptive information processing, and case conceptualization. J. EMDR Pract. Res. 1, 68–87. 10.1891/1933-3196.1.2.68

[B128] Shapiro F. Laliotis D. (2015). EMDR therapy for trauma-related disorders, in Evidence Based Treatments for Trauma-Related Psychological Disorders, eds Schnyder U. Cloitre M. (Zurich: Springer), 205–228.

[B129] Shapiro F. Maxfield L. (2002). Eye Movement Desensitization and Reprocessing (EMDR): information processing in the treatment of trauma. J. Clin. Psychol. 58, 933–946. 10.1002/jclp.10068 12115716

[B130] Sharpley C. F. Montgomery I. M. Scalzo L. A. (1996a). An investigation of some hypothetical mechanisms underlying EMDR. Scand. J. Behav. Ther. 25, 87–98. 10.1080/16506079609456013

[B131] Sharpley C. F. Montgomery I. M. Scalzo L. A. (1996b). Comparative efficacy of EMDR and alternative procedures in reducing the vividness of mental images. Scand. J. Behav. Ther. 25, 37–42.

[B132] Shepherd J. Stein K. Milne R. (2000). Eye movement desensitization and reprocessing in the treatment of post-traumatic stress disorder: a review of an emerging therapy. Psychol. Med. 30, 863–871. 10.1017/S0033291799002366 11037095

[B133] Smeets M. A. Dijs M. W. Pervan I. Engelhard I. M. van den Hout M. A. (2012). Time-course of eye movement-related decrease in vividness and emotionality of unpleasant autobiographical memories. Memory 20, 346–357. 10.1080/09658211.2012.665462 22537073

[B134] Sondergaard H. P. Elofsson U. (2008). Psychophysiological studies of EMDR. J. EMDR Pract. Res. 2, 282–288. 10.1891/1933-3196.2.4.282

[B135] Stickgold R. (2002). EMDR: a putative neurobiological mechanism of action. J. Clin. Psychol. 58, 61–75. 10.1002/jclp.1129 11748597

[B136] Stickgold R. (2008). Sleep-dependent memory processing and EMDR action. J. EMDR Pract. Res. 2, 289–299.

[B137] Stickgold R. Wehrwein P. (2009). Sleep now, remember later. Newsweek 153, 56–57. 19418973

[B138] Taylor S. Thordarson D. S. Maxfield L. Fedoroff I. C. Lovell K. Ogrodniczuk J. (2003). Comparative efficacy, speed, and adverse effects of three PTSD treatments: exposure therapy, EMDR, and relaxation training. J. Consult. Clin. Psychol. 71, 330–338. 10.1037/0022-006X.71.2.330 12699027

[B139] Tesarz J. Leisner S. Gerhardt A. Janke S. Seidler G. H. Eich W.. (2014). Effects of eye movement desensitization and reprocessing (EMDR) treatment in chronic pain patients: a systematic review. Pain Med. 15, 247–263. 10.1111/pme.12303 24308821

[B140] Thomaes K. Engelhard I. M. Sijbrandij M. Cath D. C. Van den Heuvel O. A. (2016). Degrading traumatic memories with eye movements: a pilot functional MRI study in PTSD. Eur. J. Psychotraumatol. 7:31371. 10.3402/ejpt.v7.3137 27906119 PMC5131454

[B141] van den Berg D. P. de Bont P. A. van der Vleugel B. M. de Roos C. de Jongh A. Van Minnen A.. (2015a). Prolonged exposure vs eye movement desensitization and reprocessing vs waiting list for posttraumatic stress disorder in patients with a psychotic disorder: a randomized clinical trial. JAMA Psychiatry 72, 259–267. 10.1001/jamapsychiatry.2014.2637 25607833

[B142] van den Berg D. P. de Bont P. A. van der Vleugel B. M. de Roos C. de Jongh A. van Minnen A.. (2015b). Trauma-focused treatment in PTSD patients with psychosis: symptom exacerbation, adverse events, and revictimization. Schizophr. Bull. 42, 693–702. 10.1093/schbul/sbv172 26609122 PMC4838096

[B149] van den Hout M. Muris P. Salemink. E. Kindt M. (2001). Autobiographical memories become less vivid and emotional after eye movements. Br. J. Clin. Psychol. 40, 121–130. 10.1348/014466501163571 11446234

[B143] van den Hout M. A. Bartelski N. Engelhard I. M. (2013). On EMDR: eye movements during retrieval reduce subjective vividness and objective memory accessibility during future recall. Cogn. Emot. 27, 177–183. 10.1080/02699931.2012.691087 22765837

[B144] van den Hout M. A. Eidhof M. B. Verboom J. Littel M. Engelhard I. M. (2014). Blurring of emotional and non-emotional memories by taxing working memory during recall. Cogn. Emot. 28, 717–727. 10.1080/02699931.2013.848785 24199660

[B145] van den Hout M. A. Engelhard I. M. Beetsma D. Slofstra C. Hornsveld H. Houtveen J.. (2011a). EMDR and mindfulness. Eye movements and attentional breathing tax working memory and reduce vividness and emotionality of aversive ideation. J. Behav. Ther. Exp. Psychiatry 42, 423–431. 10.1016/j.jbtep.2011.03.004 21570931

[B147] van den Hout M. A. Engelhard I. M. Rijkeboer M. M. Koekebakker J. Hornsveld H. Leer A.. (2010). Counting during recall: Taxing of working memory and reduced vividness and emotionality of negative memories. Appl. Cogn. Psychol. 24, 303–311. 10.1002/acp.167725855820

[B146] van den Hout M. A. Engelhard I. M. Rijkeboer M. M. Koekebakker J. Hornsveld H. Leer A.. (2011b). EMDR: eye movements superior to beeps in taxing working memory and reducing vividness of recollections. Behav. Res. Ther. 49, 92–98. 10.1016/j.brat.2010.11.003 21147478

[B148] van den Hout M. A. Rijkeboer M. M. Engelhard I. M. Klugkist I. Hornsveld H. Toffolo M. J.. (2012). Tones inferior to eye movements in the EMDR treatment of PTSD. Behav. Res. Ther. 50, 275–279. 10.1016/j.brat.2012.02.001 22440458

[B150] Van Loey N. E. Van Son M. J. (2003). Psychopathology and psychological problems in patients with burn scars: epidemiology and management. Am. J. Clin. Dermatol. 4, 245–272. 10.2165/00128071-200304040-00004 12680803

[B151] van Schie K. Engelhard I. M. van den Hout M. A. (2015). Taxing working memory during retrieval of emotional memories does not reduce memory accessibility when cued with reminders. Front. Psychiatry 6:16. 10.3389/fpsyt.2015.00016 25729370 PMC4325664

[B152] van Schie K. van Veen S. C. Engelhard I. M. Klugkist I. van den Hout M. A. (2016). Blurring emotional memories using eye movements: individual differences and speed of eye movements. Eur. J. Psychotraumatol. 7:29476. 10.3402/ejpt.v7.29476 27387843 PMC4933794

[B153] van Veen S. C. Engelhard I. M. van den Hout M. A. (2016). The effects of eye movements on emotional memories: using an objective measure of cognitive load. Eur. J. Psychotraumatol. 7:30122. 10.3402/ejpt.v7.30122 27387845 PMC4933790

[B154] van Veen S. C. van Schie K. Wijngaards-de Meij L. D. Littel M. Engelhard I. M. van den Hout M. A. (2015). Speed matters: relationship between speed of eye movements and modification of aversive autobiographical memories. Front. Psychiatry 6:45. 10.3389/fpsyt.2015.00045 25904871 PMC4387929

[B155] Weingarten C. P. Strauman T. J. (2015). Neuroimaging for psychotherapy research: current trends. Psychother. Res. 25, 185–213. 10.1080/10503307.2014.883088 24527694 PMC4135014

[B156] Wilson D. L. Silver S. M. Covi W. G. Foster S. (1996). Eye movement desensitization and reprocessing: effectiveness and autonomic correlates. J. Behav. Ther. Exp. Psychiatry 27, 219–229. 10.1016/S0005-7916(96)00026-2 8959423

[B157] Wolpe J. (1954). Reciprocal inhibition as the main basis of psychotherapeutic effects. Ama Arch. Neurol. Psychiatry 72, 205–226. 10.1001/archneurpsyc.1954.02330020073007 13180056

[B158] Yaggie M. Larry S. Seth M. Angela A. Chad W. Mike G. (2016). Electroencephalography coherence, memory vividness, and emotional valence effects of bilateral eye movements during unpleasant memory recall and subsequent free association: implications for eye movement desensitization and reprocessing. J. Emdr Pract. Res. 9, 79–97. 10.1891/1933-3196.9.2.78

